# Genome-wide SNP identification in *Prunus* rootstocks germplasm collections using Genotyping-by-Sequencing: phylogenetic analysis, distribution of SNPs and prediction of their effect on gene function

**DOI:** 10.1038/s41598-020-58271-5

**Published:** 2020-01-30

**Authors:** Verónica Guajardo, Simón Solís, Rubén Almada, Christopher Saski, Ksenija Gasic, María Ángeles Moreno

**Affiliations:** 1 Centro de Estudios Avanzados en Fruticultura (CEAF), Rengo, Chile; 2 Department of Plant and Environmental Sciences, Clemson University, Clemson, SC 29634 USA; 3 Department of Pomology, Estación Experimental de Aula Dei-CSIC, 50059 Zaragoza, Spain

**Keywords:** Agricultural genetics, Plant breeding

## Abstract

Genotyping-by-Sequencing (GBS) was applied in a set of 53 diploid *Prunus* rootstocks and five scion cultivars from three subgenera (*Amygdalus*, *Prunus* and *Cerasus*) for genome-wide SNP identification and to assess genetic diversity of both Chilean and Spanish germplasm collections. A group of 45,382 high quality SNPs (MAF >0.05; missing data <5%) were selected for analysis of this group of 58 accessions. These SNPs were distributed in genic and intergenic regions in the eight pseudomolecules of the peach genome (Peach v2.0), with an average of 53% located in exonic regions. The genetic diversity detected among the studied accessions divided them in three groups, which are in agreement with their current taxonomic classification. SNPs were classified based on their putative effect on annotated genes and KOG analysis was carried out to provide a deeper understanding of the function of 119 genes affected by high-impact SNPs. Results demonstrate the high utility for *Prunus* rootstocks identification and studies of diversity in *Prunus* species. Also, given the high number of SNPs identified in exonic regions, this strategy represents an important tool for finding candidate genes underlying traits of interest and potential functional markers for use in marker-assisted selection.

## Introduction

*Prunus* is a genus belonging to the subfamily *Prunoideae* of the family *Rosaceae*^1^. Several species of this large genus, known as stone fruits, are among the most important for the world fruit industry, providing edible and tasty fruits highly appreciated by consumers (e.g., peaches, plums, cherries, apricots and almonds). Cherries and plums are well adapted to the cooler temperate areas of the world, while almonds, apricots and peaches are grown in warmer temperate regions with Mediterranean climate. Nevertheless, all of them require adequate winter chilling to achieve an effective fruit set and production^2^.

In modern stone fruit production, trees require to be grafted onto a rootstock well adapted to the soil most prevalent conditions. It means that the tree is composed of two genetically distinct partners joined by grafting of the aerial part (scion) on the rootstock, the later including part of the trunk and roots. The scion is the fruiting cultivar while the rootstock is responsible for water and nutrient uptake. In addition, some of the most important agricultural traits of the tree may be substantially influenced by the rootstock^3–6^, such as blossom, fruit set, size, sugars and other fruit quality parameters, as well as tolerance to biotic and abiotic stresses. While new stone fruit scion cultivars development is addressed by numerous breeding programs around the world, only very few of them aim to the development of new *Prunus* rootstocks.

In Chile, the ‘Centro de Estudios Avanzados en Fruticultura’ (CEAF) started a *Prunus* rootstock breeding program in 2010, with collaboration of the ‘Estación Experimental de Aula Dei-Consejo Superior de Investigaciones Científicas’ (EEAD-CSIC). From 1950, the survey and establishment of Spanish *Prunus* germplasm collections were conducted by EEAD-CSIC aiming to preserve and use this material in breeding programs to obtain new stone fruit rootstocks, with specific adaptation to Mediterranean environments^7^. Furthermore, new rootstocks generated by CEAF and EEAD-CSIC have the potential to be graft-compatible with scions from different species^8^, which is a desirable characteristic for stone fruit producers. Therefore, better understanding of the molecular background of the material currently released by and/or used in rootstock breeding programs is of great importance.

Effective utilization of *Prunus* rootstocks in breeding programs depends upon accurate and unambiguous characterization^9^. In addition, the knowledge of the genetic diversity and relationships among the cultivated species of *Prunus* is important to recognize gene pools, to identify pitfalls in germplasm collections, and to develop effective conservation and management strategies^2^. Current trends in breeding stone fruit rootstocks are based on the production of interspecific hybrids, aiming at combining favorable traits from different species^7,10^. Genetic characterization of diversity and relationships at the interspecific level include studies on the systematic relationships within *Prunus* using allozyme polymorphisms^11^, chloroplast DNA variation^12–14^, Internal Transcribed Spacer (ITS) sequence variation of nuclear ribosomal DNA^15^, ITS and chloroplast *trnL*–*trnF* spacer sequence variation^16^ and Amplified Fragment Length Polymorphisms (AFLPs)^2^. Molecular characterizations and estimation of relationship specifically between *Prunus* rootstocks have been performed using molecular markers, such as Random Amplification of Polymorphic DNA (RAPD)^17,18^ and Single Sequence Repeats (SSRs)^9,19–21^. In recent years, advances in next-generation sequencing (NGS) have enabled the use of Single-Nucleotide Polymorphisms (SNPs) as other important type of molecular marker.

In the last decade, SNPs have become the markers of choice in molecular genetics due to their frequency in genomes and high-throughput, cost effectiveness for their detection using various approaches and platforms^22,23^. SNPs have been indicated as the major factors in the creation of phenotypic variation and their effect on functional changes of genes is used as a tool in functional genomics of organisms^24^. For *Prunus* species, the availability of the peach genome sequence^25,26^ enables anchoring of SNPs identified through NGS to corresponding positions in the genome, identification of SNP-carrying genes and prediction of the effect of SNPs.

Genotyping-by-Sequencing (GBS) is one of the NGS approaches, which enables the simultaneous discovery and genotyping of thousands of SNPs in a set of multiplexed samples^27^. In this approach, single and/or double-digestion of DNA with restriction enzymes is used to produce a reduced representation of the genome of each sample^27,28^. Single and/or double-digest GBS generate massive datasets of SNPs for a range of applications and is widely applicable in both model and non-model organisms^29–31^. It has been described that, compared with single-digest GBS, double-digest protocol greatly simplifies quantification of the library prior to sequencing and could generate a suitable and uniform complexity reduction of the genome^28^.

In *Prunus* species, single-digest GBS has been used for identification of a high number of SNPs for linkage maps construction^32–35^ and analysis of population genetic structure^36–38^. The use of double-digest GBS in *Prunus* has not been reported as far as the authors know. Also, and despite their importance, molecular characterization of *Prunus* rootstocks using GBS or other NGS tools has not been published. In this work, we used double-digest GBS for the identification of SNPs from 58 diploid accessions, most of them rootstocks and interspecific hybrids, members of three different *Prunus* subgenera (*Amygdalus*, *Prunus* and *Cerasus*, plus *Prunus*-*Amygdalus* hybrids). Some of these accessions are representatives of the most important breeding programs of the world (released during the last two decades), such as INRA (France) and UC Davis (USA) programs, and they are extensively used by the fruit industry. Genetic relationship between accessions, the prediction of SNP effects and the identification of SNP-carrying genes is presented. The usefulness of the presented information in understanding the genomic and phenotypic differences among *Prunus* accessions and its potential towards substantial improvement in knowledge about the genome structure of accessions from *Prunus* subgenera is discussed.

## Results

### High-throughput genotyping of *Prunus* accessions

Double-digest GBS produced between 6,801,412 and 14,293,180 read pairs, with an average of 11,117,746 reads per individual. This extremely deep sequencing led to a mean depth per SNP of 1,323 across the entire dataset (Supplementary Table S1). The number of unique tags varied among 2,174,744 and 4,952,765, with an average of 3,651,464 tags per individual. A total of 45,382 high quality SNPs (MAF > 0.05; missing data < 5%), evenly distributed over the eight pseudomolecules of peach (Pp01 to Pp08), were identified. The number of identified SNPs ranged from 4,122 for Pp08 to 10,762 for Pp01 (Table 1). A total of 224.5 Mb (99.4%) of the peach genome was covered with marker density of approximately one SNP per 5 Kb. Gaps were observed in all pseudomolecules, with the largest gap per pseudomolecule ranging from 456 Kb (Pp06) to 1.3 Mb (Pp04). Physical position of each SNP along peach pseudomolecules allowed the identification of common markers with the cherry 6 K SNP array v1^39^ and IRSC 9 K peach SNP array v1^40^ that were updated with the Peach v2.0 as a reference genome (www.rosaceae.org)^41^. Only a group of 49 SNPs were in common between our study and the cherry 6 K SNP array v1, while 75 SNPs were common between our study and the IRSC 9 K peach SNP array v1 (data not shown).

**Table 1:** Physical position of SNPs detected by Genotyping-by-Sequencing for 58 *Prunus* accessions from subgenera *Amygdalus*, *Cerasus* and *Prunus*, as well as hybrids between these subgenera.

Pseudomolecule	Size (bp)	No. SNPs	Physical position of the first SNP (bp)	Physical position of the last SNP (bp)	Scaffold coverage (bp)	Scaffold coverage (%)	Scaffold coverage (bp)/No. SNPs	Maximum gap (bp)	Last SNP before the maximum gap	First SNP after the maximum gap
**Pp01**	47,851,208	10,762	60,526	47,767,955	47,707,429	99.7	4,433.0	820,485	20,222,054	21,042,539
**Pp02**	30,405,870	5,464	202,123	30,328,437	30,126,314	99.1	5,513.6	1,124,185	12,233,140	13,357,325
**Pp03**	27,368,013	5,014	80,888	27,263,572	27,182,684	99.3	5,421.4	839,584	11,883,898	12,723,482
**Pp04**	25,843,236	5,153	66,757	25,842,651	25,775,894	99.7	5,002.1	1,264,812	18,802,344	20,067,156
**Pp05**	18,496,696	4,385	140,775	18,446,896	18,306,121	99.0	4,174.7	951,113	6,809,932	7,761,045
**Pp06**	30,767,194	5,963	76,624	30,724,506	30,647,882	99.6	5,139.7	455,992	21,314,594	21,770,586
**Pp07**	22,388,614	4,519	1,825	22,319,659	22,317,834	99.7	4,938.7	918,858	4,329,583	5,248,441
**Pp08**	22,573,980	4,122	81,834	22,513,105	22,431,271	99.4	5,441.8	956,262	7,430,911	8,387,173
**Total**	225,694,811	45,382			224,495,429					
**Average**						99.4	5,008.1			

**Figure 1:**
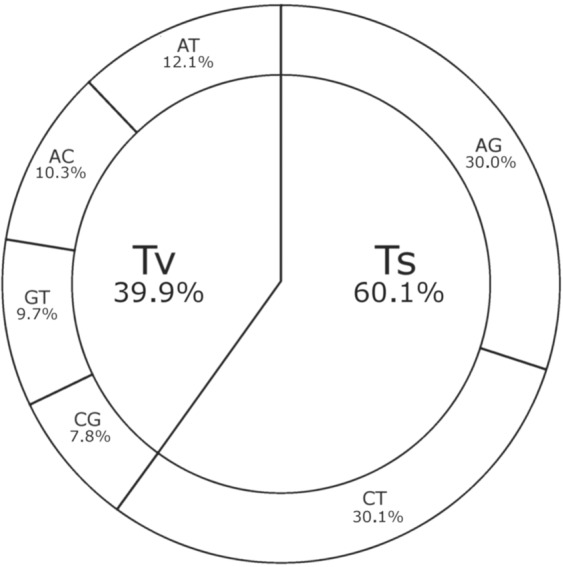
Classification of SNPs based on their nucleotide substitutions, either transitions (Ts) or transversions (Tv).

Based on the nucleotide substitution, SNPs were classified into transitions (Ts) and transversions (Tv) (Fig. 1). Transitions were observed in 27,236 (60.1%) and transversions in 18,146 SNPs (39.9%). The frequency of substitutions was 13,639 (30.1%) C/T, 13,597 (30.0%) A/G, 5,497 (12.1%) A/T, 4,697 (10.3%) A/C, 4,394 (9.7%) G/T, and 3,558 (7.8%) C/G, with the transitions to transversions ratio of 1.5.

**Figure 2:**
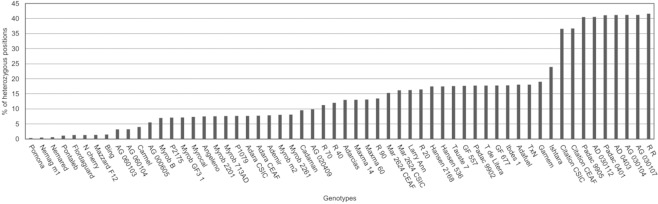
Percentage of heterozygous positions for 58 *Prunus* accessions used in the study. Mar 2624 CEAF - ‘Mariana 2624’ CEAF; Mar 2624 CSIC - ‘Mariana 2624’ CSIC; Myrob 713AD – ‘Myrobalan 713AD’; Myrob 2201 – ‘Myrobalan 2201’; Myrob 2261 – ‘Myrobalan 2261’; Myrob B – ‘Myrobalan B’; Myrob GF3-1 – ‘Myrobalan GF3-1’; Myrob m2 – ‘Myrobalan m2’; N cherry – Nanking cherry; Nemag m1 – ‘Nemaguard’ m1; R 20 – ‘Densipac’; R 40 – ‘Nanopac’; R 70 – ‘Purplepac’; R 90 – ‘Greenpac’; R R – ‘Replantpac’; T de Litera – ‘Tamarite de Litera’; T × N – ‘Titan × Nemared’.

Percentage of heterozygous positions is presented in Fig. 2 and Supplementary Table S2. Accessions showing the lowest percentage of heterozygous sites were ‘Pomona’ (0.35%), ‘Nemaguard’ (0.51%) and ‘Nemared’ (0.61%), all members of the *Amygdalus* subgenus, while those showing the highest percentage of heterozygous sites were the *Prunus-Amygdalus* hybrids AG-‘030104’ (41.22%), ‘AG-030107’ (41.23%) and ‘R R’ (41.64%). The two duplicates of ‘Adara’ and ‘Citation’ shared similar results, while the two ‘Mariana 2624’ samples were not concordant.

### Phylogenetic analysis

**Figure 3:**
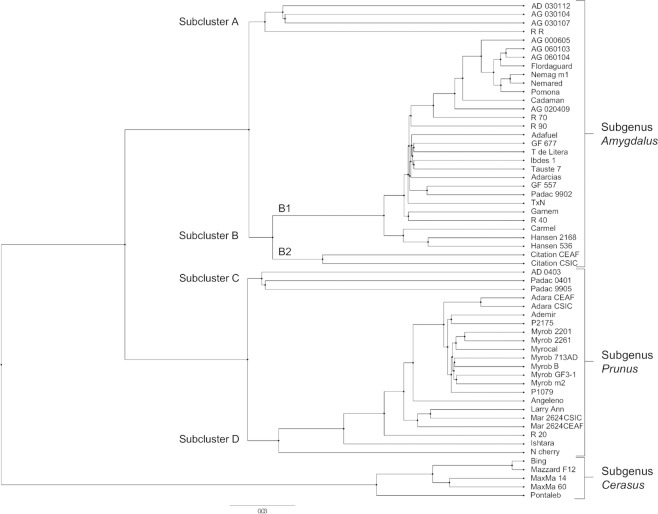
Phylogenetic analysis of 58 *Prunus* accessions generated through the UPGMA method. Mar 2624 CEAF - ‘Mariana 2624’ CEAF; Mar 2624 CSIC - ‘Mariana 2624’ CSIC; Myrob 713AD – ‘Myrobalan 713AD’; Myrob 2201 – ‘Myrobalan 2201’; Myrob 2261 – ‘Myrobalan 2261’; Myrob B – ‘Myrobalan B’; Myrob GF3-1 – ‘Myrobalan GF3-1’; Myrob m2 – ‘Myrobalan m2’; N cherry – Nanking cherry; Nemag m1 – ‘Nemaguard’ m1; R 20 – ‘Densipac’; R 40 – ‘Nanopac’; R 70 – ‘Purplepac’; R 90 – ‘Greenpac’; R R – ‘Replantpac’; T de Litera – ‘Tamarite de Litera’; T × N – ‘Titan × Nemared’.

An UPGMA dendrogram grouped accessions into three major clusters, which, in most cases, were in agreement with their taxonomic classification (subgenera *Amygdalus*, *Prunus* and *Cerasus;* Table 2 and Fig. 3). Cluster *Amygdalus* consisted of 31 accessions divided in two subclusters, one formed by four accessions (Subcluster A) and other formed by 27 accessions (Subcluster B). Subcluster A was comprised of four accessions, all *Prunus-Amygdalus* hybrids. Subcluster B is divided in two groups, B1, with 25 accessions, and B2, with two accessions. Group B1 is further divided in two subgroups, with ‘Carmel’ (*P. dulcis*), and ‘Hansen 2168’ and ‘Hansen 536’ (both *P. dulcis* × *P. persica* hybrids) being separated from the main subgroup. All accessions in the main subgroup have *P. persica* in their genetic background. Accessions with *P. davidiana* in their genetic background were grouped more tightly, separated from accessions with *P. dulcis* in their background. Group B2 is formed by two samples of ‘Citation’, a *Prunus*-*Amygdalus* hybrid (*P. salicina* × *P. persica*), one from CEAF and the other one from CSIC.

**Table 2:** Description of accessions and rootstock material used in this study.

Rootstock	Species	Origin^d^
**Subgenus** ***Amygdalus***		
Adafuel^a^	*P. dulcis* × *P. persica*	CSIC, Spain
Adarcias^a^	*P. dulcis* × *P. persica*	CSIC, Spain
AG-000605^b^	*(P. persica* × *P. davidiana)* × *P. persica*	AI, Spain
AG-020409^b^	*P. persica* × *(P. dulcis* × *P. persica)*	AI, Spain
AG-060103^b^	*P. persica* × *(P. persica* × *P. davidiana)*	AI, Spain
AG-060104^b^	*P. persica* × *(P. persica* × *P. davidiana)*	AI, Spain
Cadaman^a^	*P. persica* × *P. davidiana*	INRA, France-Hungary
Carmel^c^	*P. dulcis*	U.S.A.
Flordaguard^a^	*P. persica* × *P. davidiana*	UF, U.S.A.
Garnem^a^	*P. persica* × *P. dulcis*	CITA, Spain
GF-577^a^	*P. dulcis* × *P. persica*	INRA, France
GF-677^a^	*P. dulcis* × *P. persica*	INRA, France
Greenpac (R 90)^a^	*(P. persica* × *P. davidiana)* × *(P. dulcis* × *P. persica)*	AI, Spain
Hansen 536^a^	*P. dulcis* × *P. persica*	UC, U.S.A.
Hansen 2168^a^	*P. dulcis* × *P. persica*	UC, U.S.A.
Ibdes 1^b^	*P. dulcis* × *P. persica*	CSIC, Spain
Nanopac (R 40)^a^	*(P. dulcis* × *P. persica)* × *(P. dulcis* × *P. persica)*	AI, Spain
Nemaguard m1^a^	*P. davidiana* × *P. persica*	USDA, U.S.A.
Nemared^a^	*(P. persica* × *P. davidiana)* × *P. persica*	USDA, U.S.A.
PADAC 99-02^b^	*(P. dulcis* × *P. persica)* × *(P. dulcis* × *P. davidiana)*	AI, Spain
Pomona^c^	*P. persica*	NI
Purplepac (R 70)^a^	*(P. persica* × *P. davidiana)* × *(P. dulcis* × *P. persica)*	AI, Spain
Tamarite de Litera (T. de Litera)^b^	*P. dulcis* × *P. persica*	CSIC, Spain
Tauste 7^b^	*P. dulcis* × *P. persica*	CSIC, Spain
Titan × Nemared (T × N)^b^	*P. dulcis* × *P. persica*	U.S.A.
**Subgenus** ***Cerasus***		
Bing^c^	*P. avium*	U.S.A.
Maxma 14^a^	*P. mahaleb* × *P. avium*	U.S.A.
Maxma 60^a^	*P. mahaleb* × *P. avium*	U.S.A.
Mazzard F12/1^a^	*P. avium*	EM, England
Nanking cherry (N. cherry)^b^	*P. tomentosa*	NI
Pontaleb^a^	*P. mahaleb*	INRA, France
**Subgenus** ***Prunus***		
Adara CEAF^a^	*P. cerasifera*	CSIC, Spain
Adara CSIC^a^	*P. cerasifera*	CSIC, Spain
Ademir^a^	*P. cerasifera*	CSIC, Spain
Angeleno^c^	*P. salicina*	SWI, U.S.A.
Densipac (R 20)^a^	*P. besseyi* × *P. cerasifera*	AI, Spain
Larry Ann^c^	*P. salicina*	U.S.A.
Mariana 2624 CSIC^a^	*P. cerasifera* × *P. munsoniana*	U.S.A.
Mariana 2624 CEAF^a^	*P. cerasifera* × *P. munsoniana*	U.S.A.
Myrobalan 713 AD^b^	*P. cerasifera*	CSIC, Spain
Myrobalan 2201^b^	*P. cerasifera*	NI
Myrobalan 2261^b^	*P. cerasifera*	NI
Myrobalan GF 3-1^a^	*P. cerasifera* × *P. salicina*	INRA, France
Myrobalan B^a^	*P. cerasifera*	EM, U.K.
Myrobalan m2^b^	*P. cerasifera*	NI
Myrocal^a^	*P. cerasifera*	INRA, France
P1079^b^	*P. cerasifera*	INRA, France
P2175^b^	*P. cerasifera*	INRA, France
***Prunus-Amygdalus*** **hybrids**		
AD 030112^b^	*P. cerasifera* × *P. persica*	CSIC, Spain
AD 04-03^b^	*P. cerasifera* × *(P. persica* × *P. dulcis)*	CSIC, Spain
AG 030104^b^	*P. cerasifera* × *P. persica*	AI, Spain
AG 030107^b^	*P. cerasifera* × *P. persica*	AI, Spain
Citation CEAF^a^	*P. salicina* × *P. persica*	ZG, U.S.A.
Citation CSIC^a^	*P. salicina* × *P. persica*	ZG, U.S.A.
Ishtara^a^	*(P. cerasifera* × *P. salicina)* × *(P. cerasifera* × *P. persica)*	INRA, France
PADAC 04-01^b^	*P. cerasifera* × *(P. persica* × *P. dulcis)*	CSIC, Spain
PADAC 99-05^b^	*P. cerasifera* × *(P. persica* × *P. dulcis)*	CSIC, Spain
Replantpac (R R)^a^	*P. cerasifera* × *P. dulcis*	AI, Spain

^a^commercial rootstocks; ^b^pre-breeding materials or local *Prunus* germplasm used in *Prunus* rootstocks breeding programs; ^c^commercial scion cultivars; ^d^AI = Agromillora Iberia S.L., private nursery, Spain; CITA = Centro de Investigación y Tecnología Agroalimentaria de Aragón; CSIC = Consejo Superior de Investigaciones Científicas; EM = East Malling Research Station; INRA = Institut National de la Recherche Agronomique; SWI = Sun World International; UC = University of California; UF = University of Florida; USDA = U. S. Department of Agriculture; ZG = Zaiger Genetics; NI: no information available.

Cluster *Prunus* is comprised of 22 accessions, divided in Subcluster C, with three accessions, and Subcluster D, with 19 accessions. Subcluster C is formed by *Prunus-Amygdalus* hybrids [*P. cerasifera* × (*P. persica* × *P. dulcis*)]. In the Subcluster D, accessions with *P. cerasifera* in their genetic background are grouped separately from other accessions. Two unexpected results were observed in the Subcluster D, with ‘Larry Ann’ not grouping with ‘Angeleno’, both *P. salicina*, but instead grouping with ‘Mariana 2624’ (*P. cerasifera* × *P. munsoniana*) accessions. Also, ‘Mariana 2624’ accessions from both CEAF and CSIC were clustered together, but results suggest that they are not the same accessions. Two interspecific hybrids, ‘R 20’ (*P. besseyi* × *P. cerasifera*) and ‘Isthara’ [(*P. cerasifera* × *P. salicina*) × (*P. cerasifera* × *P. persica*)], were grouped in this subcluster. Nanking cherry (*P. tomentosa*) was also grouped in the Subcluster D, although it was expected to be grouped with accessions from subgenus *Cerasus*.

Cluster *Cerasus* was comprised of five accessions, with ‘Bing’ and ‘Mazzard F12/1’ (*P. avium*) being closely clustered and separated from ‘Pontaleb’ (*P. mahaleb*). ‘Maxma 14’ and ‘Maxma 60’, interspecific hybrids considered to be of *P. mahaleb* × *P. avium* parentage, were grouped with *P. avium* accessions.

### Population structure analysis

**Figure 4:**
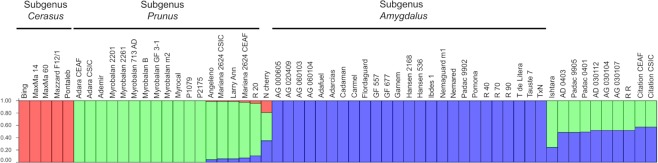
Estimation of the population structure for 58 *Prunus* accessions from subgenera *Amygdalus*, *Cerasus* and *Prunus*, as well as hybrids between these subgenera. Vertical bars along the horizontal axis represent accessions classified in their estimated membership in each population with the optimal population number *K* = 3. Three different colors represent different populations, which are related with three *Prunus* subgenera, *Amygdalus* (blue), *Prunus* (green) and *Cerasus* (red). N cherry – Nanking cherry; R 20 – ‘Densipac’; R 40 – ‘Nanopac’; R 70 – ‘Purplepac’; R 90 – ‘Greenpac’; R R – ‘Replantpac’; T de Litera – ‘Tamarite de Litera’; T × N – ‘Titan × Nemared’. A group of accessions with colored segments, indicates their admixed origin.

Population genetic structure among the studied *Prunus* accessions suggested the maximum ∆K-value of *K* = 3 (Fig. 4), as seen in phylogenetic analysis (Fig. 3). The structure analysis grouped 25 accessions in one population (*Amygdalus*), 17 in the second population (*Prunus*) and five in the third population (*Cerasus*). Eleven accessions were classified as admixed and nine of these accessions showed approximately 50% of membership to both *Prunus* and *Amygdalus* populations (Supplementary Table S3). As expected, ‘Ishtara’ showed 76% of membership to the *Prunus* population and 24% to the *Amygdalus* population. An interesting result was observed for Nanking cherry, which showed 45% of membership to *Prunus*, 36% to *Amygdalus* and 19% to *Cerasus*. The two duplicates samples of ‘Mariana 2624’ showed a different percentage of membership (90% for ‘Mariana 2624’ CEAF and 93% for ‘Mariana 2624’ CSIC), while the duplicates of ‘Adara’ and ‘Citation’ were identical.

### Principal components analysis

**Figure 5:**
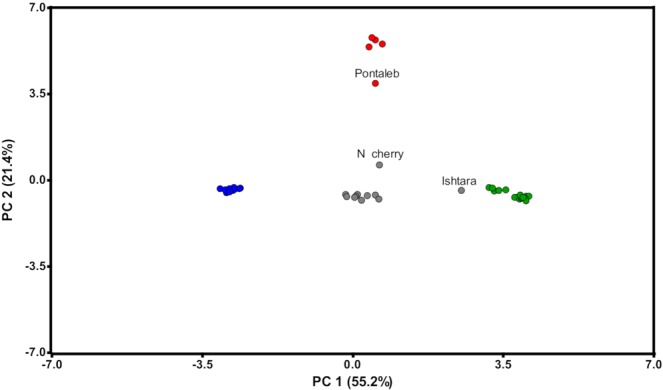
Principal components analysis (PCA) plot using 45,382 SNPs for 58 *Prunus* accessions. First and second principal components are shown and the proportion of the variance explained by each principal component is indicated in parenthesis. Colors refer to *K* = 3 genetic populations following Structure analyses; *Amygdalus* (blue); *Prunus* (green); *Cerasus* (red); admixed accessions (gray). N cherry – Nanking cherry.

The first two components of a principal component analysis (PCA) described 55.2% and 21.4% of the variance, respectively. Results supported phylogenetic (Fig. 3) and population structure analyses (Fig. 4). A cluster of *Prunus*-*Amygdalus* accessions was located in the central area of the PC1 and PC2 plot, and the *Amygdalus*, *Prunus* and *Cerasus* clusters diverged from it (Fig. 5). Three accessions are individually positioned and separated from these groups: ‘Pontaleb’ (*P. mahaleb*), in the vicinity of cluster *Cerasus*; ‘Ishtara’, near members of subgenus *Prunus*; and Nanking cherry, in the middle, closer to the cluster *Prunus* than to the cluster *Cerasus*.

### Classification of SNPs based on the positions on the peach genome

Detailed analysis of the classification of SNPs along the eight peach pseudomolecules was performed using data from only 55 unique accessions; one of the two samples of the accessions analyzed in duplicate was included in the study; e.g., ‘Adara’ CSIC, ‘Citation’ CEAF and ‘Mariana 2624’ CSIC.

**Figure 6:**
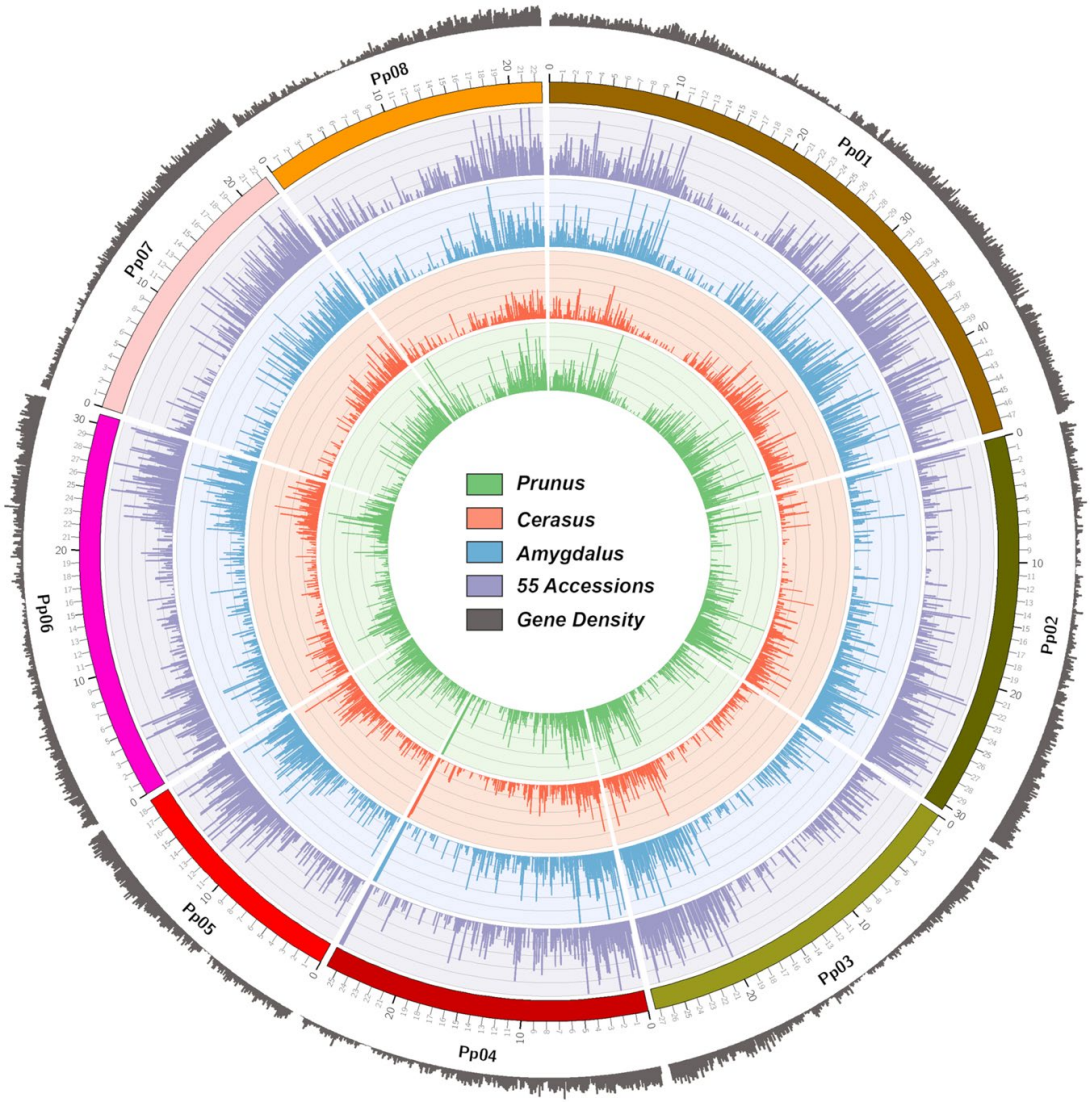
SNP distribution and gene density on the Peach v2.0 pseudomolecules. Gene and SNP density were plotted in 1 Mbp sliding window using Circos. Tracks from outside to inside are: distribution of genes on peach genome sequence; pseudomolecules of peach; and four histogram circles of SNPs distribution for 55 *Prunus* accessions (purple), 25 accessions from *Amygdalus* subgenus (blue), 5 from *Cerasus* subgenus (red) and 15 from *Prunus* subgenus (green).

SNP location and gene density (gray) along the eight pseudomolecules of the peach genome, visualized in Circos plot, are presented in Fig. 6. SNP density was determined for the whole group of 55 accessions (purple) and for each subgenus (*Amygdalus* in blue, *Cerasus* in red and *Prunus* in green). *Prunus*-*Amygdalus* hybrids and Nanking cherry were included only in the analysis of 55 accessions. A non-uniform pattern of SNP distribution along the eight pseudomolecules was observed when all 55 accessions were considered as well as when accessions within a subgenus were considered. SNP distribution along pseudomolecules was correlated with gene distribution.

**Figure 7:**
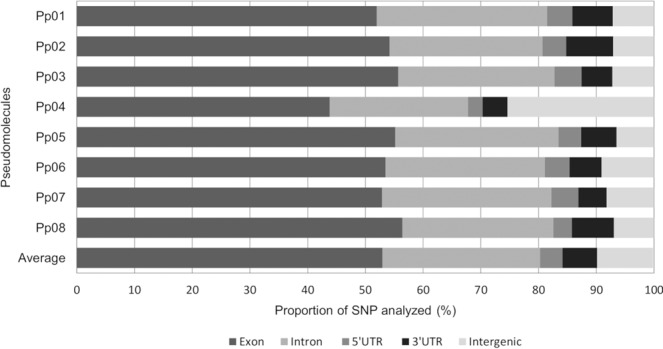
Distribution of SNPs in genic [exonic, intronic, 5′- and 3′- untranslated (UTR)] and intergenic regions using the physical position of each SNP on Peach v2.0^26^. Figure represents coverage of each SNP category per pseudomolecule and an average of all categories across all pseudomolecules.

From the initial set of 45,382 SNPs identified, 4,302 (9.7%) were detected in intergenic regions (Fig. 7 and Supplementary Table S4). Proportion of SNPs located in the intergenic regions ranged from 6.5% in Pp05 to 25.3% in Pp04, with 1,647 SNPs (corresponding to 38.3% of SNPs in intergenic regions) located in putative promoter regions (considering 1,000 bp upstream of the transcription start site; Supplementary Table S5). The majority of SNPs (41,080 SNPs, 90.3%) were located in genic regions (53.0% in exons, 27.3% in introns, 4.0% in 5′UTR and 6.0% in 3′UTR). Distribution of SNPs located in exons varied between 43.8–56.4% in Pp04 and Pp08, respectively; in introns between 24.0% for Pp04 and 29.5% for Pp01; in 5′UTR regions between 2.5% for Pp04 and 4.7% for Pp03; and in 3′UTR region between 4.4% for Pp04 and 8.2% for Pp02 (Fig. 7 and Supplementary Table S4). The lowest number of SNPs in genic regions (74.7%) was observed on Pp04, while the highest (93.5%) was observed on Pp05. The 41,080 of SNPs located in genic sequences were present in 4,884 different genes (or 18.2% of genes identified in the peach genome sequence), with an average of 8.4 variants per gene. No significant differences were found when the percentage of SNPs located in genic and intergenic regions for each subgenus were compared to each other and with those identified for the group of 55 accessions (Supplementary Table S6).

### SNP effect prediction

Classification of 45,382 SNPs based on their putative effect on annotated genes, predicted most of the SNPs with a modifier effect (76.0% of the SNPs with impact on noncoding regions); followed by SNPs with a low (14% of the SNPs with synonymous substitution); moderate (9.9% of the SNPs could have a non-synonymous substitution); and high impact (0.1% of the SNPs with disruptive impact on the protein). SNPs with a modifier effect had a downstream gene variant, with a default length of 5 kbp downstream of the most distal polyA addition site, as the most frequent variant (39.0%) (Supplementary Table S7). The most frequent mutations for SNPs with a low effect were synonymous variants (91.4%), while missense variants (99.1%) were the most frequent mutation within SNPs with a moderate effect. Finally, SNPs with a high impact had stop gained variants as the most frequent mutation (57.8%).

**Figure 8:**
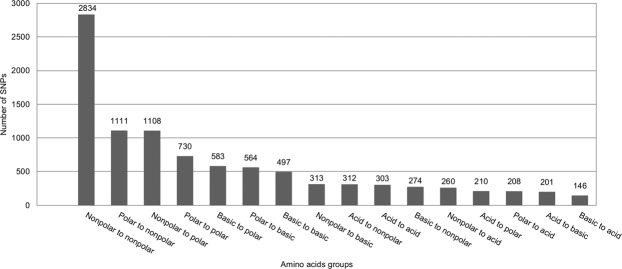
Substitution patterns of the properties of amino acid residues caused by SNPs.

SNPs classified as having moderate and high impact were further analyzed in more detail. The missense variant of the SNPs with moderate effect were classified according to the predicted changes in the charges or polarity of the amino acid residues they could cause (Fig. 8). Nonpolar to nonpolar substitutions, which do not alter the properties of amino acid residues, were the most frequent variant. Polar to nonpolar and nonpolar to polar substitutions, which cause changes in polarity, were the second and third more frequent variants, respectively. Less frequent substitutions were basic to acid variations, which are related with changes in charges of amino acid residues.

**Figure 9:**
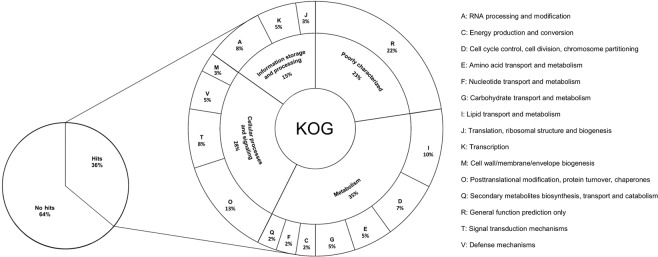
KOG-annotation-based classification of genes harboring high impact SNPs.

A group of 128 SNPs with potential to generate a high impact on gene function and phenotype of the plant material analyzed in this study were identified (Supplementary Tables S8 and S9). This group of SNPs affected 119 genes in the peach genome, with at least one SNP in most of the genes, except Prupe.1G243700, Prupe.1G433200, Prupe.2G030900, Prupe.2G230500, Prupe.5G138700, Prupe.6G273700 and Prupe.8G211800, which had two, and Prupe.5G026300, with three SNPs (Supplementary Table S8). A group of 94 SNPs was present in coding DNA sequences (exons), 29 in introns, two in 5′UTR and three in 3′UTR. The most frequent predicted effect caused by SNPs with a high impact was stop gained (72 SNPs), followed by splice donor and acceptor variants (43 SNPs in total) and stop lost (12 SNPs). Details about genes affected by high impact SNPs are presented in Supplementary Table S8. The variations observed in 55 accessions caused by 128 high impact SNPs are presented in Supplementary Table S9, including the pseudomolecule where each SNP is located, the physical position on the pseudomolecule, the gene affected by the SNP, location of the SNP (exon, intron, etc.) and the change observed using the peach genome as reference. For most of these SNPs, accessions from the same subgenus exhibited the same genotype which is either matching the reference or not. For example, in Prupe.1G152100 *Amygdalus* accessions had the same SNP as the reference (C/C), while *Prunus* and *Cerasus* accessions show the variation (T/T). Out of the 119 genes, only 36% had hits in KOG database (Fig. 9). Most of the genes were associated with metabolism (35%), with lipid transport and metabolism as the most frequent function, followed by genes related with cellular processes and signaling (28%), and information storage and processing (15%), while 23% of these genes were poorly characterized.

## Discussion

The discovery and identification of genomic variants such as SNPs, together with the determination of their location in the genome, can provide valuable information for breeding programs. In plants, many traits of interest have been linked with SNPs^23,42,43^ and these linkages have been exploited to understand individual variation, community diversity, and the evolution of species^44^. In this study, we conducted deep Genotyping-by-Sequencing and genome-wide SNP discovery on a diverse collection of accessions representing three different subgenera of the genus *Prunus* (*Amygdalus*, *Prunus* and *Cerasus*). This included 53 diploid *Prunus* rootstocks and five scion cultivars. Some of the accessions included in this work are extensively used by the stone fruit industry around the world. In fact, millions of trees on these rootstocks are sold and planted every year and they remain in the orchard for 10–20 years, depending on the fruit species. That makes this research relevant through time and to broad audience, and the results provide valuable insight into genetic diversity and relationships at the interspecific level within *Prunus*.

SNPs identified from GBS have been previously used in *Prunus* for phylogenetic and population structure analysis in apricot^37^ using 90 accessions of different origins and DNA digestion using *ApeK*I restriction enzyme. Single-digest GBS was also used for phylogenetic analysis using 11 Japanese plum cultivars to explore their natural allelic diversity in relation to the most important phenology events and fruit quality traits^38^. In this work, we used a double-digest strategy (*Pst*I/*Msp*I) and deep coverage paired-end sequencing for genome-wide representation of each *Prunus* sample. In our experience and in other published reports, paired-end sequence reads can be more accurately mapped onto the reference genome when compared to single-end reads^45^, which results in a significantly greater number of quality SNPs and lower amounts of missing data.

The availability of a reference genome sequence allows for a positional reference for each SNP for genome-wide analysis. The group of SNPs identified in the present study could prove useful for various marker-based applications in *Prunus*. In fact, the genus *Prunus* is well enabled with the availability of several reference genome sequences^25,26,46–49^. However, it must be noted that each genome is prone to harbor insertions and deletions with respect to genomes from other species. For example, in the first work published using GBS, Elshire *et al*.^27^ observed that BLAST results indicated that the majority of non-aligning reads represented maize sequences that were absent in the reference genome version used for the analysis. In peach, some genes have been reported as mutated when they were compared with similar sequences from other *Prunus* species^50–54^. Nevertheless, in spite of these differences, results obtained in this study further corroborate the high degree of synteny found among *Prunus* species reported previously^34,55–57^. Even though our analysis included species from three different *Prunus* subgenera, we observed an exceptionally high read mapping rate to the reference genome.

In comparison to previous studies where genotypes were collected by a community SNP array, only a group of 49 SNPs were in common with the cherry 6 K SNP array v1^39^, and 75 SNPs with the IRSC 9 K peach SNP array v1^40^. This could be explained by technical differences in the two genotyping approaches where peach SNP array does not contain A/T nor C/G SNPs, for example, and because the accessions used for SNP identification are different. Also, GBS allows for discovery of the SNPs that are different between the genotyped samples and the reference genome, therefore reflecting the difference between peach genome sequence and the material analyzed. Among newly discovered SNPs, transitions were more frequent than transversions which was previously observed in different plant species^22,58–60^. This is to be expected because transitions are less likely to result in amino acid substitutions and are therefore more likely to persist as silent substitutions in populations. The *Amygdalus* subgenus exhibited the lowest percentage of heterozygous SNPs, while *Prunus*-*Amygdalus* hybrids had the highest number of heterozygous SNPs. This is logical when the reference genome is considered, where material closer to *P. persica* is less heterozygous, e.g. ‘Pomona’, a pure *P. persica*. Other accessions with a low percentage of heterozygous SNPs, ‘Pontaleb’, ‘Mazzard F12/1’ and ‘Bing’, belong to *Cerasus* subgenus. These results could be explained with variations in the extent of genetic diversity across stone fruit species, ranging from a narrow genetic base in peaches; intermediate for apricots, sweet cherries and sour cherries; and higher genetic variability in almonds and plums^61,62^.

Classification of 58 *Prunus* accessions into three major genetically distinct groups was consistent with their expected pedigree relationships and parentage, which is in agreement with previous studies featuring accessions useful as rootstocks for different *Prunus* species^9,18,19,21^. Classification of hybrids between *Prunus* and *Amygdalus*, e.g. ‘Ishtara’, clearly reflected their origin with the proportion of genetic content belonging to both *Prunus* and *Amygdalus* subgenus. An interesting result was observed for Nanking cherry (*P. tomentosa*), classified as a member of the section *Microcerasus* within the subgenus *Cerasus*^1^ or the subgenus *Prunus*^2,11,63^. The *P. tomentosa* accession was grouped within *Prunus* in our study, although the structure results reveal similarities with all three subgenera (*Amygdalus*, *Prunus* and *Cerasus*). Mowrey and Werner^11^ suggested *P. tomentosa* being more primitive than other *Prunus* species, which could explain our results.

Detailed analysis of the physical position of each SNP detected in 55 accessions revealed a non-uniform pattern of SNP distribution in all eight peach pseudomolecules, related to gene density along pseudomolecules (Fig. 6). Similar distribution has been observed in *Solanum*^44^ using SNP from ESTs of *Solanum habrochaites* and *S. lycopersicum*. In the peach genome, most of the regions with low number of SNPs were associated with the putative position of the centromere of each pseudomolecule^26^. One exception to the SNP density being associated with gene density is the bottom region of pseudomolecule 4, which had high number of SNPs in our material, but low gene density (Figs. 6, 7 and Supplementary Table S4).

The association between single nucleotide change and gene function has been reported for a number of traits^44,59,64–67^. Therefore, the identification of non-synonymous SNPs would be biologically meaningful, and useful for functional genomics, molecular genetics, and marker-assisted selection in breeding. Our results revealed that SNPs located in genic regions (90.3% of the total group) were identified in 4,884 genes in the peach genome, with 53% of SNPs located in exons. The number of SNPs located in genic regions is higher than previously observed in two sweet cherry cultivars and their progeny^34^, where 65.5% of SNPs were located in genic regions and 49.8% were located in exonic regions. Differences could be due to the use of different restriction enzymes, different version of the peach genome (*ApeKI* and Peach v1.0 in the previous work and *PstI*/*MspI* and Peach v2.0 in this work) and material analyzed. Despite the differences in the methodology between the studies, the percentage of SNPs located in exon regions was similar, with less SNPs identified in intergenic regions and higher number of SNPs located in intron and 5′- and 3′-UTR in this study. This information might be useful for future applications because SNPs in the upstream, downstream, and 5′- and 3′- UTR regions might affect transcription and/or translation. However, the actual SNP effects have to be confirmed on case-by-case basis^68^ because not all mutations are functionally important and different proteins and domains differ in how well they tolerate mutations^69^.

As expected, the SNPs classified as modifier in our study were more abundant that the other categories, while the high impact SNPs were the smaller proportion. The same was observed in other crops, for example bean^70^, soybean^71^ and pear^72^. In contrast, the SNPs with impact on protein efficiency and loss-of-function, that have a direct impact on gene function with adaptive interference during the course of selection, were reported in a smaller proportion. To the best of our knowledge, this is the first work using members from different subgenera which were analyzed using GBS and where SNPs were classified according to their putative effect on annotated genes. Consequently, it was not possible to compare our results with those from other related works. Similar distribution of SNPs in respect to their putative effect was observed in a collection of bean accessions^70^ by using the Diversity Arrays Technology methodology (DArT), also based on genome complexity reduction using restriction enzymes (*PstI*/*MseI*) and SNP detection through hybridization of PCR fragments^73^.

The group of 128 high impact SNPs identified in this study, which are located in 119 genes, could have a direct effect on the gene functionality in the group of accessions analyzed. These SNPs caused either stop codon gain, splice donor and acceptor variants or stop codon loss. By performing KOG analysis (Fig. 9 and Supplementary Table S8) of these genes to investigate their putative functional class, it was observed that no functional class could be assigned to a larger fraction (64%) of them and they have been annotated as hypothetical proteins in the peach genome. However, among the genes with predicted class, those involved in metabolism were the most abundant. Our analysis allowed to identify an important group of genes affected in three *Prunus* subgenera, with nucleotide changes observed for some subgenus and not for others. Detailed analyses are needed, but this information will facilitate investigation of the consequences of predicted SNPs and their biological role. Involvement of SNPs in genes reported to be playing a role in metabolism, cellular processes and signaling, and information storage and processing could be addressed in detail at breeding level.

## Conclusion

The 45,382 GBS-derived SNPs identified in this study represent a valuable resource for molecular characterization of commercial and selected *Prunus* rootstocks. This resource provides foundation for analysis of the genetic diversity among the different interspecific hybrids and species in the germplasm collections of CEAF and EEAD-CSIC for their conservation, management and utilization in current or future rootstock breeding programs. Informative SNPs identified in this study, particularly in coding and non-coding regulatory sequence components of various genes, once validated, can be utilized as potential markers in genetic and association mapping for identifying major trait-regulatory candidate genes/QTLs in *Prunus*.

## Material and Methods

### Plant material

The group of 58 diploid accessions used in this study (Table 2) are part of two *Prunus* rootstock collections: 33 accessions coming from CEAF in Chile and 25 from EEAD-CSIC in Spain. These accessions belong to three *Prunus* subgenera: *Amygdalus* (n = 25), *Prunus* (n = 17), *Cerasus* (n = 6) and 10 hybrids between subgenera *Prunus* and *Amygdalus*. Detailed information about accessions is provided in Table 2. To compare results from GBS, three commercial rootstocks (‘Adara’, ‘Citation’ and ‘Mariana 2624’) were analyzed in duplicate, with one sample from each rootstock collection.

### DNA extraction and quantification

For genomic DNA extraction, young leaves of each accession were collected and stored at −80 °C until use. The DNeasy Plant Mini kit (Qiagen) and the NucleoSpin Plant II kit (Macherey-Nagel) were used for DNA extraction for samples from CEAF and EEAD-CSIC, respectively, according to the manufacturer’s instructions. DNA quality was examined by 1% agarose gels and DNA quantity was determined by spectrophotometry (Tecan Tradind AG, Switzerland).

### Genotyping-by-Sequencing

GBS was carried out at Clemson University Genomics Computational Laboratory (CUGCL; Clemson, SC, USA). A reduced representation GBS library was prepared using restriction enzymes *PstI* (methylation sensitive) and *MspI* (partial sensitivity to methylation), as described by Poland *et al*.^28^ in cereals. A total of 200 ng of intact genomic DNA was digested and ligated to custom designed adapter sequences. A total of 58 GBS libraries were sequenced on an Illumina HiSeq2500 using a 2x125 bp paired-end read module across 2 high-output lanes. Raw sequence data was demultiplexed and preprocessed for errors using the Stacks demultiplex tool^74^. Sample specific sequences were aligned to the eight pseudomolecules representing the eight chromosomes of the peach genome assembly (Peach v2.0)^26^ with the GMAP/GSNAP release 816.16^75^. The resulting variant call file (.vcf) was filtered for SNPs with a minimum depth (DP) of six, and present in at least 80% of the accessions. Mean coverage of each GBS SNP was determined by creating a.BED file from the final SNP set and generating a bed graph with the genomecov function of bedtools v. 2.28.0^76^, and intersecting the bedgraph with the SNP.bed file with the intersect function in bedtools. The mean coverage of each sample was determined with in house scripts. SNPs were extracted using the GBS pipeline implemented in TASSEL 5.2.5 software^77^ and accessions were called using minor allele frequency (MAF) > 0.05.

### SNPs analysis

SNPs were labeled according to the pseudomolecules in the peach genome (Pp01 to Pp08), followed by the physical position in base pairs (bp). Location of each SNP within genic [exonic, intronic, and untranslated regions (UTR)] and intergenic regions was determined using a custom Perl script (www.perl.org) with Peach v2.0 as reference. The physical position of each SNP was used to identify common markers among this study, the RosBREED cherry 6 K SNP array v1^39^ and the IRSC 9 K peach SNP array v1^40^.

Transitions/transversions and percentage of heterozygous positions were determined using SNiPlay3 (http://sniplay.southgreen.fr)^78^.

### Phylogenetic and population structure analysis

An UPGMA dendrogram was constructed using Archeopteryx software within TASSEL^77^. In order to identify population structure, the SNP genotyping information was analyzed with the program STRUCTURE v2.3.4^79^. Analysis were carried out for a range of *K* values from 1 to 10, with 10 runs for each *K*. A burn-in of 5,000 and 50,000 MCMC replications were implemented for each run. The optimal number of *K* clusters was estimated using the Δ*K* parameter of Evanno *et al*.^79^ in Structure Harvester (http://taylor0.biology.ucla.edu/structureHarvester/)^80^. Accessions were subdivided into different populations according to their maximum membership probability among the populations and the membership probabilities threshold of 0.80. Furthermore, principal components analysis (PCA) was performed on genotype scores using the PCA function in TASSEL and visualized using Infostat v2017^81^ to confirm population structure among accessions.

### Functional characterization of SNPs

Circos software v 0.69–3^82^ was used to plot the histograms of both gene and SNP density for each pseudomolecule of the peach genome sequence^26^. Gene and SNP density were assessed and plotted in a window of 1000/kb. In a first analysis, SNP density was determined for 55 accessions (considering only one of the replicates of ‘Adara’, ‘Citation’ and ‘Mariana 2624’). In a second analysis, accessions were separated by subgenus (25 accessions from *Amygdalus* subgenus, 15 from *Prunus* and 5 from *Cerasus*; Table 2). *Prunus*-*Amygdalus* hybrids and Nanking cherry were just considered for the first analysis of 55 accessions. A Chi-square test was performed to compare the SNP distribution in genic (exonic, intronic and 5′- and 3′-UTR) and intergenic regions for the three subgenus (*Amygdalus*, *Prunus* and *Cerasus*) and for the group of 55 accessions analyzed in this study.

Prediction of SNP effects was performed using SnpEff v 4.3e^68^ based on the *P. persica* gene annotation (www.rosaceae.org). Whenever multiple transcripts for a gene exist, the effect on each transcript was analyzed. The SNP predicted effects were categorized by impact, as modifier (with impact on noncoding regions), low (synonymous substitution); moderate (non-synonymous substitution); or high (disruptive impact on the protein). To investigate the putative function of the genes containing high impact SNPs, a eukaryotic orthologous group (KOG) analysis was carried out using tools from Join Genome Institute (JGI, https://jgi.doe.gov).

## Supplementary information

Supporting Information.

## Data Availability

The dataset generated for this study is available in the NCBI-SRA database, BioProject number PRJNA489327.
