# Supplementary material for: Genome-wide SNP identification in Prunus rootstocks germplasm collections using Genotyping-by-Sequencing: phylogenetic analysis, distribution of SNPs and prediction of their effect on gene function
Source: Sci Rep. 2020 Jan 30;10:1467. doi: 10.1038/s41598-020-58271-5 (PMC6992769; doi:10.1038/s41598-020-58271-5)
Supplement: Supplementary file 1 — Supporting Information. [file 41598_2020_58271_MOESM1_ESM.pdf]

Genome-wide SNP identification in *Prunus* rootstocks germplasm collections using Genotyping-by-Sequencing: phylogenetic analysis, distribution of SNPs and prediction of their effect on gene function.

Verónica Guajardo[1], Simón Solís[1], Rubén Almada[1], Christopher Saski[2], Ksenija Gasic[2], María Ángeles Moreno[3*]

[1]Centro de Estudios Avanzados en Fruticultura (CEAF), Rengo, Chile
[2]Department of Plant and Environmental Sciences, Clemson University, Clemson, SC 29634, USA
[3]Department of Pomology, Estación Experimental de Aula Dei-CSIC, 50059 Zaragoza, Spain

Suplementary Table S1. Sequencing depth, mean coverage and unique tags of rootstock GBS SNPs.

Rootstock	# of read pairs	mean depth per SNP	unique tags
AD 030112	8,095,172	744	2,836,430
AD 04-03	10,837,144	887	3,571,636
Adafuel	11,209,928	1,520	3,636,862
Adara CEAF	12,076,008	578	4,057,291
Adara CSIC	13,942,692	727	4,952,765
Adarcias	9,202,320	1,396	2,834,000
Ademir	9,732,812	904	3,151,947
AG 000605	10,541,228	3,409	3,360,523
AG 020409	11,176,184	2,532	3,817,593
AG 030104	12,292,676	1,161	4,216,679
AG 030107	13,144,904	1,698	4,375,573
AG 060103	11,265,056	1,085	4,399,585
AG 060104	13,152,980	1,123	4,753,235
Angeleno	10,743,596	586	3,651,795
Bing	10,271,048	1,029	3,268,771
Cadaman	13,428,212	2,588	3,987,738
Carmel	13,217,316	2,570	4,569,428
Citation CEAF	10,610,976	714	3,653,642
Citation CSIC	10,510,424	530	3,277,349
Flordaguard	11,595,872	1,661	3,885,901
Garnem	11,421,560	1,409	4,177,327
GF 557	9,745,244	773	3,195,017
GF 677	10,131,816	1,975	3,165,606
Hansen 2168	10,683,880	1,934	3,222,740
Hansen 536	10,338,388	1,748	3,098,486
Ibdes 1	11,288,916	1,895	3,642,863
Ishtara	11,572,792	1,011	3,833,646
Larry Ann	12,885,048	1,159	4,207,433
Mariana 2624 CEAF	13,973,044	975	4,285,291
Mariana 2624 CSIC	8,400,076	748	2,700,150
Maxma 14	10,063,628	745	3,303,350
Maxma 60	10,371,444	999	3,635,288
Mazzard F12/1	10,540,824	1,190	3,151,960
Myrobalan 2201	13,401,308	1,033	4,161,204
Myrobalan 2261	11,153,388	768	3,516,192
Myrobalan 713AD	10,603,768	744	3,297,013
Myrobalan B	9,555,752	768	2,951,473
Myrobalan GF3-1	10,171,056	744	3,346,848
Myrobalan m2	13,170,512	964	3,950,575
Myrocal	6,801,412	692	2,174,744
Nanking cherry	12,386,596	635	4,577,524
Nemaguard m1	14,293,180	3,443	4,919,243
Nemared	11,843,308	2,131	4,178,017
P1079	10,509,576	459	2,207,591
P2175	6,866,044	520	3,262,592
PADAC 04-01	10,050,780	627	2,886,950
PADAC 99-02	9,034,568	843	3,477,431
PADAC 99-05	10,935,392	697	2,204,310
Pomona	7,238,764	1,972	3,976,894
Pontaleb	11,226,692	781	4,520,796
R 20	13,174,764	1,441	3,844,236
R 40	12,745,932	2,181	4,029,608
R 70	12,238,104	2,159	4,271,698
R 90	12,496,192	2,801	3,555,677
R R	10,834,068	1,507	3,458,045
Tamarite de Litera	12,039,536	1,182	3,816,127
Tauste 7	12,269,980	1,338	3,795,866
Titan × Nemared	11,325,396	2,333	3,526,378
**Total**	644,829,276	76,761	211,784,932
**Mean**	**11,117,746**	**1,323**	**3,651,464**

Supplementary Table S2. Proportion of heterozygous sites for the group of 58 *Prunus* accessions.

Genotype	Heterozygous sites (%)
Pomona	0.35
Nemaguard m1	0.51
Nemared	0.61
Pontaleb	1.14
Flordaguard	1.34
Nanking cherry	1.36
Mazzard F12/1	1.39
Bing	1.51
AG 060103	3.22
AG 060104	3.25
Carmel	4.01
AG 000605	5.55
Myrobalan B	7.02
P2175	7.14
Myrobalan GF3-1	7.19
Myrocal	7.36
Angeleno	7.53
Myrobalan 2201	7.59
Myrobalan 713AD	7.66
P1079	7.71
Adara CSIC	7.71
Adara CEAF	7.77
Ademir	7.91
Myrobalan m2	8.04
Myrobalan 2261	8.14
Cadaman	9.56
AG 020409	9.84
R 70	11.27
R 40	12.03
Adarcias	13.00
Maxma 14	13.04
Maxma 60	13.13
R 90	13.48
Mariana 2624 CEAF	15.32
Mariana 2624 CSIC	16.18
Larry Ann	16.26
R 20	16.48
Hansen 2168	17.48
Hansen 536	17.48
Tauste 7	17.61
GF 557	17.68
PADAC 99-02	17.75
Tamarite de Litera	17.77
GF 677	17.81
Ibdes 1	17.85
Adafuel	18.06
Titan × Nemared	18.08
Garnem	19.05
Ishtara	23.93
Citation CSIC	36.61
Citation CEAF	36.74
PADAC 99-05	40.52
AD 030112	40.55
PADAC 04-01	41.11
AD 04-03	41.20
AG 030104	41.22
AG 030107	41.23
R R	41.64

Supplementary Table S3. Inferred ancestry of *Prunus* accessions determined by STRUCTURE.

	Population		
**Genotype**	***Cerasus***	***Prunus***	***Amygdalus***
AD 030112	0	0.48	0.52
AD 04-03	0	0.51	0.49
AG 000605	0	0	1
AG 020409	0	0	1
AG 030104	0	0.48	0.52
AG 030107	0	0.48	0.52
AG 060103	0	0	1
AG 060104	0	0	1
Adafuel	0	0	1
Adara CEAF	0	1	0
Adara CSIC	0	1	0
Adarcias	0	0	1
Ademir	0	1	0
Angeleno	0	0.94	0.05
Bing	1	0	0
Cadaman	0	0	1
Carmel	0	0	1
Citation CEAF	0	0.43	0.57
Citation CSIC	0	0.43	0.57
Flordaguard	0	0	1
GF 557	0	0	1
GF 677	0	0	1
Garnem	0	0	1
Hansen 2168	0	0	1
Hansen 536	0	0	1
Ibdes 1	0	0	1
Ishtara	0	0.76	0.24
Larry Ann	0.01	0.92	0.06
Mariana 2624 CEAF	0.02	0.90	0.08
Mariana 2624 CSIC	0.01	0.93	0.06
Maxma 14	1	0	0
Maxma 60	1	0	0
Mazzard F12/1	1	0	0
Myrobalan 2201	0	1	0
Myrobalan 2261	0	1	0
Myrobalan 713AD	0	1	0
Myrobalan B	0	1	0
Myrobalan GF3-1	0	1	0
Myrobalan m2	0	1	0
Myrocal	0	1	0
Nanking cherry	0.19	0.45	0.36
Nemaguard m1	0	0	1
Nemared	0	0	1
P1079	0	1	0
P2175	0	1	0
PADAC 04-01	0	0.51	0.49
PADAC 99-02	0	0.00	1
PADAC 99-05	0	0.51	0.49
Pomona	0	0.00	1
Pontaleb	1	0.00	0
R 20	0.04	0.85	0.11
R 40	0	0	1
R 70	0	0	1
R 90	0	0	1
R R	0	0.49	0.52
Tamarite de Litera	0	0	1
Tauste 7	0	0	1
Titan × Nemared	0	0	1

Supplementary Table S4. Distribution of SNPs in genic (exonic, intronic and 5'- and 3'-UTR) and intergenic regions. Peach v2.0 (Verde et al., 2017) was used as reference.

	**N° SNPs in intergenic region**	**N° SNPs in intergenic region (%)**	**N° SNPs in exon**	**N° SNPs in exon (%)**	**N° SNPs in intron**	**N° SNPs in intron (%)**	**N° SNPs in 5'UTR**	**N° SNPs in 5'UTR (%)**	**N° SNPs in 3'UTR**	**N° SNPs in 3'UTR (%)**	**Total SNPs in genic region**	**Total SNPs in genic region (%)**	**TOTAL SNPs**
**Pp01**	768	7.1	5,594	52.0	3,173	29.5	472	4.4	755	7.0	9,994	92.9	10,762
**Pp02**	386	7.1	2,962	54.2	1,445	26.4	225	4.1	446	8.2	5,078	92.9	5,464
**Pp03**	361	7.2	2,793	55.7	1,357	27.1	235	4.7	268	5.3	4,653	92.8	5,014
**Pp04**	1,306	25.3	2,258	43.8	1,235	24.0	129	2.5	225	4.4	3,847	74.7	5,153
**Pp05**	284	6.5	2,420	55.2	1,240	28.3	172	3.9	269	6.1	4,101	93.5	4,385
**Pp06**	541	9.1	3,189	53.5	1,646	27.6	254	4.3	333	5.6	5,422	90.9	5,963
**Pp07**	370	8.2	2,390	52.9	1,326	29.3	210	4.6	223	4.9	4,149	91.8	4,519
**Pp08**	286	6.9	2,325	56.4	1,079	26.2	132	3.2	300	7.3	3,836	93.1	4,122
**TOTAL**	**4,302**		**23,931**		**12,501**		**1,829**		**2,819**		**41,080**		**45,382**
**Average**		**9.7**		**53.0**		**27.3**		**4.0**		**6.0**		**90.3**	

Supplementary Table S5. SNPs located in intergenic regions, putativelly located in promotor regions considering 1,000 bp before 5'UTR.

Pseudomolecule	N° SNPs in intergenic region	SNPs located until 1000 bp before 5'UTR	SNPs located until 1000 bp before 5'UTR (%)
**Pp01**	768	361	47.0
**Pp02**	386	162	42.0
**Pp03**	361	193	53.5
**Pp04**	1,306	225	17.2
**Pp05**	284	162	57.0
**Pp06**	541	267	49.4
**Pp07**	370	183	49.5
**Pp08**	286	94	32.9
**Total**	**4,302**	**1,647**	**38.3**

Supplementary Table S6. Distribution of SNPs in genic (exonic, intronic and 5'- and 3'-UTR) and intergenic regions for three subgenus (*Amygdalus*, *Prunus* and *Cerasus*) and for the group of 55 accessions analyzed in this study. Peach v2.0 (Verde et al., 2017) was used as reference.

	p-value = 0.9801 NS	p-value = 0.9835 NS	p-value = 0.9997 NS	p-value = 0.9850 NS	p-value = 0.9992 NS
**Subgenus**	**N° SNPs in exon (%)**	**N° SNPs in intron (%)**	**N° SNPs in 5'UTR (%)**	**N° SNPs in 3'UTR (%)**	**N° SNPs in intergenic region (%)**
Amygdalus	54.06	26.54	3.81	5.81	9.78
Prunus	56.85	24.87	3.90	5.19	9.19
Cerasus	56.30	25.02	4.10	4.97	9.61
**General**	52.96	27.30	3.97	6.10	9.68

Supplementary Table S7. SNP effect prediction based on their putative effect on annotated genes.

SNPs effect classification	Percentage (%)
**MODIFIER**	
Downstream gene variant	39.0
Upstream gene variant	30.5
Intron variant	19.0
3'UTR variant	5.0
Intergenic region	4.1
5'UTR variant	2.5
**LOW**	
Synonymous variant	91.4
Splice region variant and intron variant	5.3
5'UTR premature start codon gain variant	2.0
Splice region variant and synonymous variant	0.9
Splice region variant	0.4
Stop retained variant	0.1
**MODERATE**	
Missense variant	99.1
Missense variant and splice region variant	0.9
**HIGH**	
Stop gained	57.8
Splice donor variant and intron variant	17.5
Splice acceptor variant and intron variant	14.3
Stop lost	9.1
Stop gained and splice region variant	1.3

Supplementary Table S8. Annotations of genes in which high impact SNPs were identified.

Pseud.	Physical position	Gene	Location of SNP	Ref.	Var.	Effect	Swissprot Match	Swissprot description	ID KOG	Description	Function ID	Function description	Group
Pp01	1208674	Prupe.1G017700	Intron	A/A	G/G	splice donor variant and intron variant	Q9SPL4	Vicilin-like antimicrobial peptides 2-2 OS=Macadamia integrifolia GN=AMP2-2 PE=2 SV=1	N/D				
Pp01	1518492	Prupe.1G022200	Exon	G/G	A/A	stop gained	Q3L181	Perakine reductase OS=Rauvolfia serpentina GN=PR PE=1 SV=1	KOG1575	Voltage-gated shaker-like K+ channel, subunit beta/KCNAB	C	Energy production and conversion	METABOLISM
Pp01	4233151	Prupe.1G059800	Exon	G/G	A/A	stop gained	n/a	n/a	N/D				
Pp01	5395054	Prupe.1G075300	Exon	T/T	C/C	splice donor variant and intron variant			N/D				
Pp01	10997151	Prupe.1G140000	3'UTR	T/T	C/C	splice acceptor variant and intron variant	Q9FEE2	Probable serine/threonine-protein phosphatase 2A regulatory subunit B'' subunit TON2 OS=Arabidopsis thaliana GN=TON2 PE=1 SV=1	KOG2562	Protein phosphatase 2 regulatory subunit	A	RNA processing and modification	INFORMATION STORAGE AND PROCESSING
Pp01	11643652	Prupe.1G148400	Exon	G/G	T/T	stop gained	Q9M8J3	Protein FAR1-RELATED SEQUENCE 7 OS=Arabidopsis thaliana GN=FRS7 PE=2 SV=1	N/D				
Pp01	11785551	Prupe.1G150100	Exon	G/G	T/T	stop gained	Q9LUG9	Mediator of RNA polymerase II transcription subunit 33A OS=Arabidopsis thaliana GN=MED33A PE=1 SV=1	N/D				
Pp01	12015398	Prupe.1G152100	Exon	C/C	T/T	stop gained	Q8RVH5	Basic 7S globulin 2 OS=Glycine max PE=1 SV=1	KOG1339	Aspartyl protease	O	Posttranslational modification, protein turnover, chaperones	CELLULAR PROCESSES AND SIGNALING
Pp01	24211285	Prupe.1G229100	Intron	C/C	T/T	splice donor variant and intron variant	Q92620	Pre-mRNA-splicing factor ATP-dependent RNA helicase PRP16 OS=Homo sapiens GN=DHX38 PE=1 SV=2	N/D				
Pp01	25742333	Prupe.1G243700	Exon	G/G	A/A	stop gained	Q9LYT3	Protein TRANSPARENT TESTA 12 OS=Arabidopsis thaliana GN=TT12 PE=2 SV=1	KOG1347	Uncharacterized membrane protein, predicted efflux pump	R	General function prediction only	POORLY CHARACTERIZED
Pp01	25744181	Prupe.1G243700	Intron	T/T	C/C	splice donor variant and intron variant	Q9LYT3	Protein TRANSPARENT TESTA 12 OS=Arabidopsis thaliana GN=TT12 PE=2 SV=1	KOG1347	Uncharacterized membrane protein, predicted efflux pump	R	General function prediction only	POORLY CHARACTERIZED
Pp01	26111275	Prupe.1G248600	Intron	A/A	G/G	splice donor variant and intron variant	n/a	n/a	N/D				
Pp01	29521108	Prupe.1G300600	Exon	A/A	T/T	stop gained	Q9SSQ2	F-box protein At1g52490 OS=Arabidopsis thaliana GN=At1g52490 PE=4 SV=1	N/D				
Pp01	31992246	Prupe.1G340000	Exon	C/C	T/T	stop gained	P46032	Protein NRT1/ PTR FAMILY 8.3 OS=Arabidopsis thaliana GN=NPF8.3 PE=1 SV=1	N/D				
Pp01	32289128	Prupe.1G344900	Exon	C/C	A/A	stop gained	Q9LT24	BTB/POZ domain-containing protein At3g19850 OS=Arabidopsis thaliana GN=At3g19850 PE=2 SV=1	N/D				
Pp01	32657984	Prupe.1G350900	Exon	G/G	A/A	stop gained	n/a	n/a	N/D				
Pp01	33304427	Prupe.1G361900	Intron	A/A	C/C	splice donor variant and intron variant	Q94CH6	GDSL esterase/lipase EXL3 OS=Arabidopsis thaliana GN=EXL3 PE=2 SV=1	N/D				
Pp01	33701054	Prupe.1G368300	Exon	G/G	T/T	stop gained	P93024	Auxin response factor 5 OS=Arabidopsis thaliana GN=ARF5 PE=1 SV=3	N/D				
Pp01	35498042	Prupe.1G402700	Exon	C/C	T/T	stop gained	Q8GXZ3	Serine/threonine-protein kinase At5g01020 OS=Arabidopsis thaliana GN=At5g01020 PE=1 SV=1	KOG1187	Serine/threonine protein kinase	T	Signal transduction mechanisms	CELLULAR PROCESSES AND SIGNALING

Pseud.	Physical position	Gene	Location of SNP	Ref.	Var.	Effect	Swissprot Match	Swissprot description	ID KOG	Description	Function ID	Function description	Group
Pp01	36138477	Prupe.1G416100	Exon	A/A	G/G	splice acceptor variant and intron variant	Q8CDF7	Exonuclease 3'-5' domain-containing protein 1 OS=Mus musculus GN=Exd1 PE=2 SV=1	N/D				
Pp01	37066369	Prupe.1G433200	Exon	A/A	T/T	stop gained	O65351	Subtilisin-like protease OS=Arabidopsis thaliana GN=ARA12 PE=1 SV=1	N/D				
Pp01	37066515	Prupe.1G433200	Exon	G/G	A/A	stop gained	O65351	Subtilisin-like protease OS=Arabidopsis thaliana GN=ARA12 PE=1 SV=1	N/D				
Pp01	37389079	Prupe.1G439800	Exon	C/C	A/A	stop gained	O48723	Patatin-like protein 2 OS=Arabidopsis thaliana GN=PLP2 PE=1 SV=1	KOG0513	Ca2+-independent phospholipase A2	I	Lipid transport and metabolism	METABOLISM
Pp01	37588240	Prupe.1G443200	Intron	T/T	C/C	stop lost	O24621	RNA polymerase sigma factor sigC OS=Arabidopsis thaliana GN=SIGC PE=2 SV=1	N/D				
Pp01	38793354	Prupe.1G464900	Exon	C/C	T/T	stop gained	Q9LUJ2	Pentatricopeptide repeat-containing protein At3g22690 OS=Arabidopsis thaliana GN=PCMP-H56 PE=2 SV=1	N/D				
Pp01	42591382	Prupe.1G518600	Intron	G/G	C/C	splice acceptor variant and intron variant	n/a	n/a	N/D				
Pp01	43540969	Prupe.1G532600	Exon	G/G	A/A	stop gained	n/a	n/a	N/D				
Pp02	3338542	Prupe.2G030900	Exon	G/G	A/A	splice acceptor variant and intron variant			N/D				
Pp02	3338543	Prupe.2G030900	Exon	A/A	G/G	splice acceptor variant and intron variant			N/D				
Pp02	4569073	Prupe.2G041600	Exon	G/G	T/T	stop gained	A7MIK3	Chaperone protein DnaJ OS=Cronobacter sakazakii (strain ATCC BAA-894) GN=dnaJ PE=3 SV=1	N/D				
Pp02	5372807	Prupe.2G046400	Exon	C/C	G/G	stop gained	Q9LRR4	Putative disease resistance RPP13-like protein 1 OS=Arabidopsis thaliana GN=RPPL1 PE=3 SV=1	KOG4658	Apoptotic ATPase	T	Signal transduction mechanisms	CELLULAR PROCESSES AND SIGNALING
Pp02	5597860	Prupe.2G048600	Intron	T/T	C/C	splice donor variant and intron variant	Q9LHG8	Protein ELC OS=Arabidopsis thaliana GN=ELC PE=1 SV=1	KOG2391	Vacuolar sorting protein/ubiquitin receptor VPS23	U	Intracellular trafficking, secretion, and vesicular transport	CELLULAR PROCESSES AND SIGNALING
Pp02	7757386	Prupe.2G061100	Exon	G/G	T/T	stop lost	P28475	NADP-dependent D-sorbitol-6-phosphate dehydrogenase OS=Malus domestica GN=S6PDH PE=2 SV=1	KOG1577	Aldo/keto reductase family proteins	R	General function prediction only	POORLY CHARACTERIZED
Pp02	16188362	Prupe.2G105000	Exon	C/C	A/A	stop gained	n/a	n/a	N/D				
Pp02	17820932	Prupe.2G120800	Exon	C/C	A/A	stop gained	Q9FNZ2	Zinc finger CCCH domain-containing protein 48 OS=Arabidopsis thaliana GN=ZFWD1 PE=2 SV=1	KOG0266	WD40 repeat-containing protein	R	General function prediction only	POORLY CHARACTERIZED
Pp02	22441824	Prupe.2G181600	Exon	G/G	T/T	stop gained	P51614	Acidic endochitinase OS=Vitis vinifera GN=CHIT3 PE=2 SV=1	N/D				
Pp02	22767709	Prupe.2G186700	Exon	G/G	A/A	splice donor variant and intron variant	O65717	Cyclic nucleotide-gated ion channel 1 OS=Arabidopsis thaliana GN=CNGC1 PE=1 SV=1	N/D				
Pp02	23077245	Prupe.2G191400	Exon	G/G	A/A	stop gained	Q67XD9	Alkaline/neutral invertase CINV2 OS=Arabidopsis thaliana GN=CINV2 PE=2 SV=1	N/D				
Pp02	23359464	Prupe.2G196200	Exon	T/T	G/G	stop lost	Q7TP47	Heterogeneous nuclear ribonucleoprotein Q OS=Rattus norvegicus GN=Syncrip PE=2 SV=1	KOG0117	Heterogeneous nuclear ribonucleoprotein R (RRM superfamily)	A	RNA processing and modification	INFORMATION STORAGE AND PROCESSING
Pp02	24535441	Prupe.2G214200	Intron	A/A	G/G	splice donor variant and intron variant	n/a	n/a	N/D				

Pseud.	Physical position	Gene	Location of SNP	Ref.	Var.	Effect	Swissprot Match	Swissprot description	ID KOG	Description	Function ID	Function description	Group
Pp02	24600784	Prupe.2G215000	Exon	A/A	T/T	splice acceptor variant and intron variant	Q9LJZ5	Ubiquitin-conjugating enzyme E2 19 OS=Arabidopsis thaliana GN=UBC19 PE=1 SV=1	KOG0421	Ubiquitin-protein ligase	O	Posttranslational modification, protein turnover, chaperones	CELLULAR PROCESSES AND SIGNALING
Pp02	25504865	Prupe.2G230500	Intron	G/G	T/T	splice donor variant and intron variant	O80638	WAT1-related protein At2g39510 OS=Arabidopsis thaliana GN=At2g39510 PE=2 SV=1	N/D				
Pp02	25504866	Prupe.2G230500	Intron	T/T	C/C	splice donor variant and intron variant			N/D				
Pp02	27301463	Prupe.2G266700	Exon	G/G	T/T	stop gained	O24496	Hydroxyacylglutathione hydrolase cytoplasmic OS=Arabidopsis thaliana GN=GLX2-2 PE=1 SV=2	KOG0813	Glyoxylase	R	General function prediction only	POORLY CHARACTERIZED
Pp02	28208092	Prupe.2G285200	Exon	T/T	A/A	stop gained	Q50EK3	Cytochrome P450 704C1 OS=Pinus taeda GN=CYP704C1 PE=2 SV=1	KOG0157	Cytochrome P450 CYP4/CYP19/CYP26 subfamilies	I	Lipid transport and metabolism	METABOLISM
Pp02	29118951	Prupe.2G305600	Exon	C/C	T/T	stop gained	Q9LYS2	ABC transporter C family member 10 OS=Arabidopsis thaliana GN=ABCC10 PE=2 SV=2	N/D				
Pp02	29778175	Prupe.2G318400	Exon	G/G	A/A	splice acceptor variant and intron variant			N/D				
Pp02	29786530	Prupe.2G318700	Exon	T/T	G/G	stop gained	n/a	n/a	KOG1267	Mitochondrial transcription termination factor, mTERF	R	General function prediction only	POORLY CHARACTERIZED
Pp02	30052808	Prupe.2G325000	Exon	T/T	G/G	stop lost	Q8RY17	Wall-associated receptor kinase-like 22 OS=Arabidopsis thaliana GN=WAKL22 PE=2 SV=1	KOG1187	Serine/threonine protein kinase	T	Signal transduction mechanisms	CELLULAR PROCESSES AND SIGNALING
Pp03	5152153	Prupe.3G071800	Exon	G/G	T/T	stop gained	P23919	Thymidylate kinase OS=Homo sapiens GN=DTYMK PE=1 SV=4	KOG3327	Thymidylate kinase/adenylate kinase	F	Nucleotide transport and metabolism	METABOLISM
Pp03	6026685	Prupe.3G081000	Exon	C/C	A/A	stop gained	n/a	n/a	N/D				
Pp03	18483519	Prupe.3G165800	Exon	C/C	G/G	stop gained	Q94JQ3	Serine hydroxymethyltransferase 3, chloroplastic OS=Arabidopsis thaliana GN=SHM3 PE=1 SV=2	N/D				
Pp03	19659004	Prupe.3G180700	Exon	A/A	T/T	stop gained	Q8RY17	Wall-associated receptor kinase-like 22 OS=Arabidopsis thaliana GN=WAKL22 PE=2 SV=1	KOG1187	Serine/threonine protein kinase	T	Signal transduction mechanisms	CELLULAR PROCESSES AND SIGNALING
Pp03	20034786	Prupe.3G185800	Exon	G/G	T/T	stop gained	n/a	n/a	N/D				
Pp03	20242526	Prupe.3G189300	Exon	C/C	A/A	stop gained	Q9ZWJ3	UDP-glycosyltransferase 85A2 OS=Arabidopsis thaliana GN=UGT85A2 PE=2 SV=1	KOG1192	UDP-glucuronosyl and UDP-glucosyl transferase	G	Carbohydrate transport and metabolism	METABOLISM
Pp03	20710092	Prupe.3G196300	Intron	C/C	A/A	splice donor variant and intron variant	n/a	n/a	N/D				
Pp03	21042678	Prupe.3G202400	Exon	A/A	T/T	stop gained	Q9LUB9	BTB/POZ domain-containing protein At5g48130 OS=Arabidopsis thaliana GN=At5g48130 PE=2 SV=1	N/D				
Pp03	22341972	Prupe.3G223400	Exon	C/C	T/T	stop gained	Q99PV0	Pre-mRNA-processing-splicing factor 8 OS=Mus musculus GN=Prpf8 PE=1 SV=2	N/D				
Pp03	22395801	Prupe.3G224500	Exon	T/T	A/A	stop gained	Q9ZR09	Putative F-box/FBD/LRR-repeat protein At4g03220 OS=Arabidopsis thaliana GN=At4g03220 PE=4 SV=1	N/D				
Pp03	22712692	Prupe.3G228000	Exon	T/T	C/C	splice acceptor variant and intron variant	Q9ZR04	Putative cyclin-D6-1 OS=Arabidopsis thaliana GN=CYCD6-1 PE=3 SV=1	N/D				

Pseud.	Physical position	Gene	Location of SNP	Ref.	Var.	Effect	Swissprot Match	Swissprot description	ID KOG	Description	Function ID	Function description	Group
Pp03	25578377	Prupe.3G279500	Exon	A/A	C/C	stop gained	Q9LFR3	Gibberellin-regulated protein 14 OS=Arabidopsis thaliana GN=GASA14 PE=1 SV=1	N/D				
Pp03	25784195	Prupe.3G284300	Exon	A/A	T/T	stop gained	O23939	2-methylene-furan-3-one reductase OS=Fragaria vesca GN=EO PE=1 SV=2	KOG1198	Zinc-binding oxidoreductase	C	Energy production and conversion	METABOLISM
Pp03	26188576	Prupe.3G292500	Exon	T/T	A/A	stop gained	Q8GWV5	U-box domain-containing protein 3 OS=Arabidopsis thaliana GN=PUB3 PE=2 SV=2	N/D				
Pp03	26460349	Prupe.3G299100	Exon	C/C	T/T	stop gained	B9SLR1	UPF0392 protein RCOM_0530710 OS=Ricinus communis GN=RCOM_0530710 PE=3 SV=1	N/D				
Pp03	26845232	Prupe.3G308000	Exon	C/C	A/A	splice acceptor variant and intron variant	n/a	n/a	N/D				
Pp03	26952573	Prupe.3G310100	Intron	A/A	C/C	stop lost	n/a	n/a	N/D				
Pp03	27263012	Prupe.3G315900	Exon	C/C	T/T	stop gained	P35614	Eukaryotic peptide chain release factor subunit 1-3 OS=Arabidopsis thaliana GN=ERF1-3 PE=2 SV=1	N/D				
Pp04	478027	Prupe.4G009700	Exon	T/T	A/A	stop gained	Q9SU29	E3 ubiquitin-protein ligase UPL5 OS=Arabidopsis thaliana GN=UPL5 PE=1 SV=1	KOG0940	Ubiquitin protein ligase RSP5/NEDD4	O	Posttranslational modification, protein turnover, chaperones	CELLULAR PROCESSES AND SIGNALING
Pp04	3230538	Prupe.4G066900	Intron	C/C	A/A	splice acceptor variant and intron variant	O14297	Uncharacterized protein C9E9.15 OS=Schizosaccharomyces pombe (strain 972 / ATCC 24843) GN=SPAC9E9.15 PE=3 SV=2	KOG1203	Predicted dehydrogenase	G	Carbohydrate transport and metabolism	METABOLISM
Pp04	4588318	Prupe.4G091900	Exon	C/C	A/A	stop gained	Q9LMP1	Wall-associated receptor kinase 2 OS=Arabidopsis thaliana GN=WAK2 PE=1 SV=1	KOG1187	Serine/threonine protein kinase	T	Signal transduction mechanisms	CELLULAR PROCESSES AND SIGNALING
Pp04	4800787	Prupe.4G095400	Exon	G/G	T/T	stop gained	n/a	n/a	N/D				
Pp04	12724300	Prupe.4G204300	Intron	A/A	G/G	splice acceptor variant and intron variant	Q6YUU5	Putative multidrug resistance protein OS=Oryza sativa subsp. japonica GN=Os02g0190300 PE=3 SV=1	N/D				
Pp04	13355976	Prupe.4G213800	Exon	C/C	A/A	stop gained	P53683	Calcium-dependent protein kinase isoform 2 OS=Oryza sativa subsp. japonica GN=CPK2 PE=2 SV=2	KOG0032	Ca2+/calmodulin-dependent protein kinase, EF-Hand protein superfamily	T	Signal transduction mechanisms	CELLULAR PROCESSES AND SIGNALING
Pp04	16671331	Prupe.4G249300	Intron	T/T	C/C	splice donor variant and intron variant	Q2KI54	Armadillo repeat-containing protein 8 OS=Bos taurus GN=ARMC8 PE=2 SV=1	KOG1293	Serine/threonine protein kinase	T	Signal transduction mechanisms	CELLULAR PROCESSES AND SIGNALING
Pp04	18519647	Prupe.4G258800	5'UTR	G/G	A/A	splice acceptor variant and intron variant	Q56YA5	Serine--glyoxylate aminotransferase OS=Arabidopsis thaliana GN=AGT1 PE=1 SV=2	KOG2862	Alanine-glyoxylate aminotransferase AGT1	R	General function prediction only	POORLY CHARACTERIZED
Pp04	21428422	Prupe.4G269200	Exon	G/G	T/T	stop gained	O74963	Nuclear control of ATPase protein 2 OS=Schizosaccharomyces pombe (strain 972 / ATCC 24843) GN=nca2 PE=3 SV=1	N/D				
Pp04	24272831	Prupe.4G283000	Exon	A/A	G/G	stop lost	C0LGT6	LRR receptor-like serine/threonine-protein kinase EFR OS=Arabidopsis thaliana GN=EFR PE=1 SV=1	KOG1187	Serine/threonine protein kinase	T	Signal transduction mechanisms	CELLULAR PROCESSES AND SIGNALING
Pp05	2977555	Prupe.5G026300	Intron	C/C	T/T	stop gained	P24521	Phosphomevalonate kinase OS=Saccharomyces cerevisiae (strain ATCC 204508 / S288c) GN=ERG8 PE=1 SV=2	KOG4519	Phosphomevalonate kinase	I	Lipid transport and metabolism	METABOLISM
Pp05	2977592	Prupe.5G026300	Intron	C/C	A/A	stop gained			KOG4519	Phosphomevalonate kinase	I	Lipid transport and metabolism	METABOLISM

Pseud.	Physical position	Gene	Location of SNP	Ref.	Var.	Effect	Swissprot Match	Swissprot description	ID KOG	Description	Function ID	Function description	Group
Pp05	2977655	Prupe.5G026300	Intron	G/G	A/A	stop gained			KOG4519	Phosphomevalonate kinase	I	Lipid transport and metabolism	METABOLISM
Pp05	9753025	Prupe.5G085800	Exon	C/C	T/T	stop gained	O65351	Subtilisin-like protease OS=Arabidopsis thaliana GN=ARA12 PE=1 SV=1	N/D				
Pp05	11156615	Prupe.5G105000	Exon	T/T	C/C	splice acceptor variant and intron variant	n/a	n/a	N/D				
Pp05	11801339	Prupe.5G115300	Exon	C/C	A/A	stop gained	n/a	n/a	KOG0978; KOG4593; KOG0963; KOG0018	E3ubiquitin ligase involved in syntaxin degradation;	O	Posttranslational modification, protein turnover, chaperones	CELLULAR PROCESSES AND SIGNALING
Pp05	12071378	Prupe.5G121100	Exon	T/T	A/A	splice donor variant and intron variant	Q5XET6	RNA pseudouridine synthase 3, mitochondrial OS=Arabidopsis thaliana GN=At1g78910 PE=2 SV=1	KOG1919	RNA pseudouridylate synthases	A	RNA processing and modification	INFORMATION STORAGE AND PROCESSING
Pp05	12483740	Prupe.5G128200	Exon	C/C	A/A	stop gained	Q6EU10	Probable N6-adenosine-methyltransferase MT-A70-like OS=Oryza sativa subsp. japonica GN=Os02g0672600 PE=2 SV=1	N/D				
Pp05	13033824	Prupe.5G138700	Exon	T/T	C/C	stop lost	A5EY31	Glutamyl-tRNA(Gln) amidotransferase subunit A OS=Dichelobacter nodosus (strain VCS1703A) GN=gatA PE=3 SV=1	KOG1211	Amidases	J	Translation, ribosomal structure and biogenesis	INFORMATION STORAGE AND PROCESSING
Pp05	13033825	Prupe.5G138700	Exon	A/A	T/T	stop lost			KOG1211	Amidases	J	Translation, ribosomal structure and biogenesis	INFORMATION STORAGE AND PROCESSING
Pp05	13052939	Prupe.5G139100	Intron	T/T	C/C	stop lost	Q39817	Calnexin homolog OS=Glycine max PE=2 SV=1	N/D				
Pp05	13822461	Prupe.5G153800	Exon	C/C	T/T	stop gained	Q8W486	Uncharacterized protein At1g04910 OS=Arabidopsis thaliana GN=At1g04910 PE=2 SV=1	N/D				
Pp05	14752314	Prupe.5G173200	Exon	G/G	T/T	stop gained	Q5UNY4	Uncharacterized protein L728 OS=Acanthamoeba polyphaga mimivirus GN=MIMI_L728 PE=4 SV=1	N/D				
Pp05	15163706	Prupe.5G181100	Exon	T/T	C/C	splice acceptor variant and intron variant	O95628	CCR4-NOT transcription complex subunit 4 OS=Homo sapiens GN=CNOT4 PE=1 SV=3	KOG2068	MOT2 transcription factor	K	Transcription	INFORMATION STORAGE AND PROCESSING
Pp06	4064429	Prupe.6G058100	Intron	G/G	A/A	splice donor variant and intron variant	Q56Y11	Dehydrodolichyl diphosphate synthase 2 OS=Arabidopsis thaliana GN=At5g58770 PE=2 SV=2	KOG1602	Cis-prenyltransferase	I	Lipid transport and metabolism	METABOLISM
Pp06	5294814	Prupe.6G077600	Exon	C/C	A/A	stop gained	Q41050	Outer envelope pore protein 16, chloroplastic OS=Pisum sativum GN=OEP16 PE=1 SV=1	N/D				
Pp06	6198291	Prupe.6G090600	Exon	A/A	T/T	stop gained	P0C7Q7	Putative pentatricopeptide repeat-containing protein At1g12700, mitochondrial OS=Arabidopsis thaliana GN=At1g12700 PE=3 SV=1	N/D				
Pp06	6626204	Prupe.6G095500	Exon	G/G	A/A	stop gained	Q9ZUY1	Pentatricopeptide repeat-containing protein At2g27800, mitochondrial OS=Arabidopsis thaliana GN=At2g27800 PE=3 SV=2	N/D				
Pp06	6776765	Prupe.6G097200	5'UTR	C/C	T/T	stop gained	Q3UZ01	RNA-binding protein 40 OS=Mus musculus GN=Rnpc3 PE=2 SV=2	N/D				
Pp06	11614585	Prupe.6G143200	Exon	G/G	T/T	stop gained	P87172	Phosphatidylinositol N-acetylglucosaminyltransferase gpi3 subunit OS=Schizosaccharomyces pombe (strain 972 / ATCC 24843) GN=gpi3 PE=3 SV=1	KOG1111	N-acetylglucosaminyltransferase complex, subunit PIG-A/SPT14, required for phosphatidylinositol biosynthesis/Sulfolipid synthase	O	Posttranslational modification, protein turnover, chaperones	CELLULAR PROCESSES AND SIGNALING

Pseud.	Physical position	Gene	Location of SNP	Ref.	Var.	Effect	Swissprot Match	Swissprot description	ID KOG	Description	Function ID	Function description	Group
Pp06	24074941	Prupe.6G242400	Exon	G/G	T/T	stop gained	P47927	Floral homeotic protein APETALA 2 OS=Arabidopsis thaliana GN=AP2 PE=1 SV=1	N/D				
Pp06	24262177	Prupe.6G245400	Exon	T/T	A/A	stop gained	Q9ZUT4	Pentatricopeptide repeat-containing protein At2g37320 OS=Arabidopsis thaliana GN=PCMP-E50 PE=2 SV=1	N/D				
Pp06	25612767	Prupe.6G269200	Intron	G/G	T/T	splice acceptor variant and intron variant	Q304B9	Neutral ceramidase OS=Arabidopsis thaliana GN=At2g38010 PE=3 SV=1	N/D				
Pp06	25865964	Prupe.6G273700	Exon	A/A	T/T	stop gained	Q9SD53	UPF0481 protein At3g47200 OS=Arabidopsis thaliana GN=At3g47200 PE=2 SV=1	N/D				
Pp06	25865968	Prupe.6G273700	Exon	C/C	A/A	stop gained			N/D				
Pp06	25907095	Prupe.6G274200	Exon	C/C	T/T	stop gained	Q9SD53	UPF0481 protein At3g47200 OS=Arabidopsis thaliana GN=At3g47200 PE=2 SV=1	N/D				
Pp06	28053849	Prupe.6G313800	Intron	T/T	C/C	splice donor variant and intron variant	P31414	Pyrophosphate-energized vacuolar membrane proton pump 1 OS=Arabidopsis thaliana GN=AVP1 PE=1 SV=1	N/D				
Pp07	355429	Prupe.7G003000	Exon	A/A	T/T	stop gained	n/a	n/a	N/D				
Pp07	1892280	Prupe.7G013300	Intron	C/C	T/T	splice acceptor variant and intron variant	Q8VXX4	Replication factor C subunit 3 OS=Arabidopsis thaliana GN=RFC3 PE=2 SV=1	KOG2035	Replication factor C, subunit RFC3	L	Replication, recombination and repair	INFORMATION STORAGE AND PROCESSING
Pp07	2597831	Prupe.7G017900	Exon	G/G	T/T	stop lost	n/a	n/a	N/D				
Pp07	3862125	Prupe.7G024500	3'UTR	G/G	A/A	splice donor variant and intron variant	P36591	Dihydrofolate reductase OS=Schizosaccharomyces pombe (strain 972 / ATCC 24843) GN=dfr1 PE=2 SV=2	KOG2551	D-arabinono-1, 4-lactone oxidase	V	Defense mechanisms	CELLULAR PROCESSES AND SIGNALING
Pp07	8234392	Prupe.7G046300	Exon	G/G	T/T	stop gained	O47881	L-galactono-1,4-lactone dehydrogenase, mitochondrial OS=Brassica oleracea PE=1 SV=1	KOG4730	D-arabinono-1, 4-lactone oxidase	V	Defense mechanisms	CELLULAR PROCESSES AND SIGNALING
Pp07	12024911	Prupe.7G085200	Exon	C/C	G/G	stop gained	Q84P24	4-coumarate--CoA ligase-like 6 OS=Arabidopsis thaliana GN=4CLL6 PE=2 SV=2	N/D				
Pp07	14919262	Prupe.7G129200	Intron	G/G	T/T	splice acceptor variant and intron variant	n/a	n/a	N/D				
Pp07	18684932	Prupe.7G199400	Intron	A/A	G/G	splice acceptor variant and intron variant	n/a	n/a	N/D				
Pp07	20336314	Prupe.7G232800	Exon	A/A	G/G	splice donor variant and intron variant	n/a	n/a	N/D				
Pp07	22201422	Prupe.7G270900	Intron	G/G	C/C	splice acceptor variant and intron variant	P55228	Glucose-1-phosphate adenylyltransferase small subunit, chloroplastic OS=Arabidopsis thaliana GN=APS1 PE=2 SV=2	KOG1322	GDP-mannose pyrophosphorylase/mannose-1-phosphate guanylyltransferase	M	Cell wall/membrane/envelope biogenesis	CELLULAR PROCESSES AND SIGNALING
Pp08	2964780	Prupe.8G031300	Exon	G/G	C/C	stop gained	n/a	n/a	N/D				
Pp08	12198711	Prupe.8G088700	Exon	A/A	T/T	splice donor variant and intron variant	P43296	Cysteine proteinase RD19a OS=Arabidopsis thaliana GN=RD19A PE=2 SV=1	N/D				
Pp08	13462058	Prupe.8G103900	Exon	C/C	G/G	stop gained	O60231	Putative pre-mRNA-splicing factor ATP-dependent RNA helicase DHX16 OS=Homo sapiens GN=DHX16 PE=1 SV=2	N/D				

Pseud.	Physical position	Gene	Location of SNP	Ref.	Var.	Effect	Swissprot Match	Swissprot description	ID KOG	Description	Function ID	Function description	Group
Pp08	14751752	Prupe.8G121500	Exon	C/C	A/A	stop gained	Q9SMM9	Probable carboxylesterase 13 OS=Arabidopsis thaliana GN=CXE13 PE=1 SV=1	KOG1515	Arylacetamide deacetylase	V	Defense mechanisms	CELLULAR PROCESSES AND SIGNALING
Pp08	16714708	Prupe.8G156100	Exon	T/T	A/A	splice donor variant and intron variant	Q93ZN9	LL-diaminopimelate aminotransferase, chloroplastic OS=Arabidopsis thaliana GN=DAP PE=1 SV=1	KOG0257	Kynurenine aminotransferase, glutamine transaminase K	E	Amino acid transport and metabolism	METABOLISM
Pp08	16744784	Prupe.8G156700	Intron	C/C	A/A	splice donor variant and intron variant	O48651	Squalene monooxygenase OS=Panax ginseng PE=2 SV=1	N/D				
Pp08	17720052	Prupe.8G174100	Intron	G/G	A/A	splice donor variant and intron variant	n/a	n/a	N/D				
Pp08	18250622	Prupe.8G183500	3'UTR	T/T	G/G	splice acceptor variant and intron variant	P93025	Phototropin-2 OS=Arabidopsis thaliana GN=PHOT2 PE=1 SV=2	KOG0610	Putative serine/threonine protein kinase	R	General function prediction only	POORLY CHARACTERIZED
Pp08	18484118	Prupe.8G189000	Exon	G/G	A/A	stop gained	Q41238	Linoleate 9S-lipoxygenase 6 (Fragment) OS=Solanum tuberosum GN=LOX1.6 PE=1 SV=1	N/D				
Pp08	19316635	Prupe.8G206200	Exon	A/A	G/G	splice donor variant and intron variant			N/D				
Pp08	19568186	Prupe.8G211800	Exon	T/T	A/A	stop lost	Q7KZ85	Transcription elongation factor SPT6 OS=Homo sapiens GN=SUPT6H PE=1 SV=2	N/D				
Pp08	19568187	Prupe.8G211800	Exon	C/C	A/A	stop lost			N/D				
Pp08	19922349	Prupe.8G218300	Exon	C/C	A/A	stop gained	Q9FNL7	Protein NRT1/ PTR FAMILY 5.2 OS=Arabidopsis thaliana GN=NPF5.2 PE=2 SV=1	N/D				
Pp08	20765179	Prupe.8G234400	Exon	A/A	T/T	stop gained	Q9M1V3	Pentatricopeptide repeat-containing protein At3g63370 OS=Arabidopsis thaliana GN=PCMP-H83 PE=2 SV=2	N/D				

Pseud. – Pseudomolecule; Ref. – Reference; Var. – Variation; ID KOG: ID from EuKaryotic Orthologous Groups

Supplementary Table S9. Variations in 55 *Prunus* accessions observed with 128 SNPs with high impact.

	**Pseudomolecule**	Pp01	Pp01	Pp01	Pp01	Pp01	Pp01	Pp01	Pp01	Pp01	Pp01	Pp01	Pp01	Pp01	Pp01	Pp01
	**Position**	1208674	1518492	4233151	5395054	10997151	11643652	11785551	12015398	24211285	25742333	25744181	26111275	29521108	31992246	32289128
	**Gene**	Prupe.1G017700	Prupe.1G022200	Prupe.1G059800	Prupe.1G075300	Prupe.1G140000	Prupe.1G148400	Prupe.1G150100	Prupe.1G152100	Prupe.1G229100	Prupe.1G243700	Prupe.1G243700	Prupe.1G248600	Prupe.1G300600	Prupe.1G340000	Prupe.1G344900
	**Location**	Intron	Exon	Exon	Exon	3'UTR	Exon	Exon	Exon	Intron	Exon	Intron	Intron	Exon	Exon	Exon
	**Reference**	A/A	G/G	G/G	T/T	T/T	G/G	G/G	C/C	C/C	G/G	T/T	A/A	A/A	C/C	C/C
	**Variation**	G/G	A/A	A/A	C/C	C/C	T/T	T/T	T/T	T/T	A/A	C/C	G/G	T/T	T/T	A/A
Amygdalus	Adafuel	A/G	G/G	G/G	T/T	T/T	G/G	G/G	C/C	C/C	G/G	T/T	A/A	A/A	C/C	C/C
	Adarcias	G/G	G/G	G/G	T/T	T/T	G/G	G/G	C/C	C/C	G/G	T/T	A/A	A/A	C/C	C/C
	AG 000605	G/G	G/G	G/G	T/T	T/T	G/G	G/G	C/C	C/C	G/G	T/T	A/A	A/A	C/C	C/C
	AG 020409	G/G	G/G	G/G	T/T	T/T	G/G	G/G	C/C	C/C	G/G	T/T	A/A	A/A	C/C	C/C
	AG 060103	G/G	G/G	G/G	T/T	T/T	G/G	G/G	C/C	C/C	G/G	T/T	A/A	A/A	C/C	C/C
	AG 060104	G/G	G/G	G/G	T/T	T/T	G/G	G/G	C/C	C/C	G/G	T/T	A/A	A/A	C/C	C/C
	Cadaman	A/G	G/G	G/G	T/T	T/T	G/G	G/G	C/C	C/C	G/G	T/T	A/A	A/A	C/C	C/C
	Carmel	A/A	G/G	G/G	T/T	T/T	G/G	T/T	C/C	C/C	G/G	T/T	A/A	N	C/C	C/C
	Flordaguard	G/G	G/G	G/G	T/T	T/T	G/G	G/G	C/C	C/C	G/G	T/T	A/A	A/A	C/C	C/C
	Garnem	A/G	G/G	G/G	T/T	T/T	G/G	G/T	C/C	C/C	G/G	T/T	A/A	A/A	C/C	C/C
	GF 557	A/G	G/G	G/G	T/T	T/T	G/G	G/T	C/C	C/C	G/G	T/T	A/A	A/A	C/C	C/C
	GF 677	A/G	G/G	G/G	T/T	T/T	G/G	G/T	C/C	C/C	G/G	T/T	A/A	A/A	C/C	C/C
	Hansen 2168	A/G	G/G	G/G	T/T	T/T	G/G	G/T	C/C	C/C	G/G	T/T	A/A	A/A	C/C	C/C
	Hansen 536	A/G	G/G	G/G	T/T	T/T	G/G	G/T	C/C	C/C	G/G	T/T	A/A	A/A	C/C	C/C
	Ibdes 1	A/G	G/G	G/G	T/T	T/T	G/G	G/T	C/C	C/C	G/G	T/T	A/A	A/A	C/C	C/C
	Nemaguard m1	G/G	G/G	G/G	T/T	T/T	G/G	G/G	C/C	C/C	G/G	T/T	A/A	A/A	C/C	C/C
	Nemared	G/G	G/G	G/G	T/T	T/T	G/G	G/G	C/C	C/C	G/G	T/T	A/A	A/A	C/C	C/C
	PADAC 99-02	A/G	G/G	G/G	T/T	T/T	G/G	G/T	C/C	C/C	G/G	T/T	A/A	A/A	C/C	C/C
	Pomona	G/G	G/G	G/G	T/T	T/T	G/G	G/G	C/C	C/C	G/G	T/T	A/A	A/A	C/C	C/C
	R 40	G/G	G/G	G/G	T/T	T/T	G/G	G/T	C/C	C/C	G/G	T/T	A/A	N	C/C	C/C
	R 70	A/A	G/G	G/G	T/T	T/T	G/G	G/T	C/C	C/C	G/G	T/T	A/A	A/A	C/C	C/C
	R 90	G/G	G/G	G/G	T/T	T/T	G/G	G/G	C/C	C/C	G/G	T/T	A/A	A/A	C/C	C/C
	Tamarite de Litera	A/G	G/G	G/G	T/T	T/T	G/G	G/T	C/C	C/C	G/G	T/T	A/A	A/A	C/C	C/C
	Tauste 7	A/G	G/G	G/G	T/T	T/T	G/G	G/T	C/C	C/C	G/G	T/T	A/A	A/A	C/C	C/C
	Titan × Nemared	A/G	G/G	G/G	T/T	T/T	G/G	G/T	C/C	C/C	G/G	T/T	A/A	A/A	C/C	C/C
Prunus	Adara CSIC	A/A	G/G	G/G	C/T	C/C	G/G	G/G	T/T	C/C	A/A	T/T	G/G	A/A	C/C	C/C
	Ademir	A/A	G/G	G/G	C/C	C/C	G/G	G/G	T/T	C/C	A/A	T/T	G/G	A/A	C/C	C/C
	Angeleno	A/A	G/G	G/G	N	N	G/G	G/G	T/T	C/C	A/A	C/C	G/G	A/A	C/C	C/C
	Larry Ann	A/A	A/G	G/G	C/T	T/T	G/G	G/G	T/T	C/C	A/A	N	G/G	A/A	C/C	C/C
	Mariana 2624 CSIC	A/A	A/G	G/G	C/T	T/T	G/G	G/G	T/T	C/C	A/A	C/T	G/G	A/A	C/C	C/C
	Myrobalan 2201	A/A	A/A	G/G	T/T	C/C	G/G	G/G	T/T	C/C	A/A	T/T	G/G	A/A	C/C	C/C
	Myrobalan 2261	A/A	A/A	G/G	C/T	C/C	G/G	G/G	T/T	C/C	A/A	T/T	G/G	A/A	C/C	C/C
	Myrobalan 713AD	A/A	A/G	G/G	N	C/C	G/G	G/G	T/T	C/C	A/A	C/T	G/G	A/A	C/C	C/C
	Myrobalan B	A/A	G/G	G/G	C/T	C/C	G/G	G/G	T/T	C/C	A/A	T/T	G/G	A/A	C/C	C/C
	Myrobalan GF3-1	A/A	A/G	G/G	C/C	C/C	G/G	G/G	T/T	C/C	A/A	T/T	G/G	A/A	C/C	C/C
	Myrobalan m2	A/A	G/G	G/G	C/T	C/C	G/G	G/G	T/T	C/C	A/A	T/T	G/G	A/A	C/C	C/C
	Myrocal	A/A	A/G	G/G	T/T	C/C	G/G	G/G	T/T	C/C	A/A	C/T	G/G	A/A	C/C	C/C
	P1079	A/A	G/G	G/G	C/C	C/C	G/G	G/G	T/T	C/C	A/A	T/T	G/G	A/A	C/C	C/C
	P2175	A/A	G/G	G/G	C/T	C/C	G/G	G/G	T/T	C/C	A/A	T/T	G/G	A/A	C/C	C/C
	R 20	A/A	A/G	G/G	C/T	C/T	G/G	G/G	T/T	C/C	A/G	T/T	G/G	A/A	C/C	C/C
Cerasus	Bing	A/A	G/G	N	T/T	N	T/T	G/G	T/T	T/T	G/G	T/T	G/G	T/T	C/C	A/A
	Maxma 14	A/A	G/G	A/A	T/T	C/C	G/T	G/G	T/T	T/T	G/G	T/T	G/G	T/T	T/T	A/A
	Maxma 60	A/A	G/G	A/A	T/T	C/C	G/T	G/G	T/T	T/T	G/G	T/T	G/G	T/T	T/T	A/A
	Mazzard F12/1	A/A	G/G	N	T/T	N	T/T	G/G	T/T	T/T	G/G	T/T	G/G	T/T	C/T	A/A
	Pontaleb	A/A	G/G	A/A	T/T	C/C	G/G	G/G	T/T	T/T	G/G	T/T	G/G	N	T/T	N
***Prunus-Amygdalus* hybrids and others**	AD 04-03	A/A	G/G	G/G	T/T	C/T	G/G	G/T	C/T	C/C	A/G	T/T	A/G	A/A	C/C	C/C
	AG 030104	A/G	G/G	G/G	C/T	C/T	G/G	G/G	C/T	C/C	A/G	T/T	A/G	A/A	C/C	C/C
	AG 030107	A/G	G/G	G/G	T/T	C/T	G/G	G/G	C/T	C/C	A/G	T/T	A/G	A/A	C/C	C/C
	AD 030112	A/G	G/G	G/G	C/T	C/T	G/G	G/G	C/T	C/C	A/G	T/T	A/G	A/A	C/C	C/C
	Citation CEAF	A/G	G/G	G/G	T/T	C/T	G/G	G/G	C/T	C/C	A/G	C/T	A/G	A/A	C/C	C/C
	Ishtara	A/A	G/G	G/G	C/C	C/C	G/G	G/G	T/T	C/C	A/A	T/T	G/G	A/A	C/C	C/C
	Nanking cherry	A/A	G/G	G/G	N	T/T	G/G	G/G	T/T	C/C	G/G	T/T	G/G	A/A	C/C	C/C
	PADAC 04-01	A/A	G/G	G/G	C/T	C/T	G/G	G/T	C/T	C/C	A/G	T/T	A/G	A/A	C/C	C/C
	PADAC 99-05	A/A	G/G	G/G	T/T	C/T	G/G	G/G	C/T	C/C	A/G	T/T	A/G	A/A	C/C	C/C
	R R	A/G	A/G	G/G	C/T	C/T	G/G	G/G	C/T	C/C	A/G	T/T	A/G	A/A	C/C	C/C

	**Pseudomolecule**	Pp01	Pp01	Pp01	Pp01	Pp01	Pp01	Pp01	Pp01	Pp01	Pp01	Pp01	Pp01	Pp02	Pp02	Pp02
	**Position**	32657984	33304427	33701054	35498042	36138477	37066369	37066515	37389079	37588240	38793354	42591382	43540969	3338542	3338543	4569073
	**Gene**	Prupe.1G350900	Prupe.1G361900	Prupe.1G368300	Prupe.1G402700	Prupe.1G416100	Prupe.1G433200	Prupe.1G433200	Prupe.1G439800	Prupe.1G443200	Prupe.1G464900	Prupe.1G518600	Prupe.1G532600	Prupe.2G030900	Prupe.2G030900	Prupe.2G041600
	**Location**	Exon	Intron	Exon	Exon	Exon	Exon	Exon	Exon	Intron	Exon	Intron	Exon	Exon	Exon	Exon
	**Reference**	G/G	A/A	G/G	C/C	A/A	A/A	G/G	C/C	T/T	C/C	G/G	G/G	G/G	A/A	G/G
	**Variation**	A/A	C/C	T/T	T/T	G/G	T/T	A/A	A/A	C/C	T/T	C/C	A/A	A/A	G/G	T/T
Amygdalus	Adafuel	G/G	A/A	G/G	C/C	A/G	A/A	G/G	C/C	T/T	C/C	G/G	G/G	G/G	A/A	G/G
	Adarcias	G/G	A/A	G/T	C/C	A/G	A/A	G/G	C/C	T/T	C/C	G/G	G/G	G/G	A/A	G/G
	AG 000605	G/G	A/A	G/G	C/C	A/A	A/A	G/G	C/C	T/T	C/C	G/G	G/G	G/G	A/A	G/G
	AG 020409	G/G	A/A	G/T	C/C	A/G	A/A	G/G	C/C	T/T	C/C	G/G	G/G	G/G	A/A	G/G
	AG 060103	G/G	A/A	G/G	C/C	A/A	A/A	G/G	C/C	T/T	C/T	G/G	G/G	G/G	A/A	G/G
	AG 060104	G/G	A/A	G/G	C/C	A/A	A/A	G/G	C/C	T/T	C/T	G/G	G/G	G/G	A/A	G/G
	Cadaman	G/G	A/A	G/G	C/C	A/A	A/A	G/G	C/C	T/T	C/C	G/G	G/G	G/G	A/A	G/G
	Carmel	G/G	N	G/T	C/C	G/G	A/A	G/G	C/C	T/T	C/C	G/G	G/G	G/G	A/A	G/G
	Flordaguard	G/G	A/A	G/G	C/C	A/A	A/A	G/G	C/C	T/T	C/T	G/G	G/G	G/G	A/A	G/G
	Garnem	G/G	A/A	G/T	C/C	A/G	A/A	G/G	C/C	T/T	C/C	G/G	G/G	G/G	A/A	G/G
	GF 557	G/G	A/A	G/G	C/C	A/G	A/A	G/G	C/C	T/T	C/T	G/G	G/G	G/G	A/A	G/G
	GF 677	G/G	A/A	G/G	C/C	A/G	A/A	G/G	C/C	T/T	C/C	G/G	G/G	G/G	A/A	G/G
	Hansen 2168	G/G	A/A	G/G	C/C	A/G	A/A	G/G	C/C	T/T	C/T	G/G	G/G	G/G	A/A	G/G
	Hansen 536	G/G	A/A	G/G	C/C	A/G	A/A	G/G	C/C	T/T	C/T	G/G	G/G	G/G	A/A	G/G
	Ibdes 1	G/G	A/A	G/T	C/C	A/G	A/A	G/G	C/C	T/T	C/C	G/G	G/G	G/G	A/A	G/G
	Nemaguard m1	G/G	A/A	G/G	C/C	A/A	A/A	G/G	C/C	T/T	C/T	G/G	G/G	G/G	A/A	G/G
	Nemared	G/G	A/A	G/G	C/C	A/A	A/A	G/G	C/C	T/T	C/C	G/G	G/G	G/G	A/A	G/G
	PADAC 99-02	G/G	A/A	G/G	C/C	A/G	A/A	G/G	C/C	T/T	C/T	G/G	G/G	G/G	A/A	G/G
	Pomona	G/G	A/A	G/G	C/C	A/A	A/A	G/G	C/C	T/T	C/C	G/G	G/G	G/G	A/A	G/G
	R 40	N	N	G/T	C/C	G/G	A/A	G/G	C/C	T/T	C/C	G/G	G/G	G/G	A/A	G/G
	R 70	G/G	A/A	G/T	C/C	A/G	A/A	G/G	C/C	T/T	C/C	G/G	G/G	G/G	A/A	G/G
	R 90	G/G	A/A	G/T	C/C	A/G	A/A	G/G	C/C	T/T	C/C	G/G	G/G	G/G	A/A	G/G
	Tamarite de Litera	G/G	A/A	G/G	C/C	A/G	A/A	G/G	C/C	T/T	C/C	G/G	G/G	G/G	A/A	G/G
	Tauste 7	G/G	A/A	G/T	C/C	N	A/A	G/G	C/C	T/T	C/C	G/G	G/G	G/G	A/A	G/G
	Titan × Nemared	G/G	A/A	G/G	C/C	A/G	A/A	G/G	C/C	T/T	C/C	G/G	G/G	G/G	A/A	G/G
Prunus	Adara CSIC	G/G	A/A	G/G	T/T	A/A	A/T	G/G	A/C	T/T	C/C	G/G	A/A	A/A	G/G	G/G
	Ademir	G/G	A/A	G/G	T/T	A/A	A/A	A/G	A/C	T/T	C/C	C/G	A/A	A/A	G/G	G/T
	Angeleno	G/G	A/A	G/G	T/T	A/A	A/A	G/G	A/C	T/T	C/C	C/G	A/A	A/A	G/G	G/G
	Larry Ann	G/G	A/C	G/G	T/T	A/A	A/A	G/G	A/C	T/T	C/C	C/G	A/G	A/A	G/G	T/T
	Mariana 2624 CSIC	G/G	A/C	G/G	T/T	N	A/T	G/G	A/C	T/T	C/C	C/G	A/G	A/A	G/G	G/T
	Myrobalan 2201	G/G	A/A	G/G	T/T	A/A	A/T	G/G	C/C	T/T	C/C	C/C	A/A	A/A	G/G	G/T
	Myrobalan 2261	G/G	A/A	G/G	T/T	A/A	A/T	G/G	A/C	T/T	C/C	C/C	A/A	A/A	G/G	G/G
	Myrobalan 713AD	G/G	A/A	G/G	T/T	A/A	A/T	G/G	A/C	T/T	C/C	C/G	A/A	A/A	G/G	G/T
	Myrobalan B	G/G	A/A	G/G	T/T	A/A	A/T	G/G	A/C	T/T	C/C	C/G	A/A	A/A	G/G	G/G
	Myrobalan GF3-1	G/G	A/A	G/G	T/T	A/A	A/T	G/G	A/C	T/T	C/C	C/C	A/A	A/A	G/G	G/G
	Myrobalan m2	G/G	A/A	G/G	T/T	A/A	A/T	G/G	A/C	T/T	C/C	C/C	A/A	A/A	G/G	G/T
	Myrocal	G/G	A/A	G/G	T/T	A/A	N	G/G	A/C	T/T	C/C	C/C	A/A	A/A	G/G	G/G
	P1079	G/G	A/A	G/G	T/T	N	A/T	G/G	A/C	T/T	C/C	C/C	A/A	A/A	G/G	G/T
	P2175	G/G	A/A	G/G	T/T	A/A	A/T	G/G	C/C	T/T	C/C	C/C	A/A	A/A	G/G	G/G
	R 20	G/G	A/C	G/G	C/T	A/A	A/T	A/G	A/C	T/T	C/C	C/G	A/G	A/A	G/G	G/G
Cerasus	Bing	A/A	A/A	G/G	C/C	A/A	A/A	G/G	C/C	C/C	C/C	N	N	A/A	G/G	G/G
	Maxma 14	A/A	A/A	G/G	C/C	A/A	A/A	G/G	C/C	C/C	C/C	G/G	G/G	A/A	G/G	G/G
	Maxma 60	A/A	A/A	G/G	C/C	A/A	A/A	G/G	C/C	C/C	C/C	G/G	G/G	A/A	G/G	G/G
	Mazzard F12/1	A/A	A/A	G/G	C/C	A/A	A/A	G/G	C/C	C/C	C/C	N	N	A/A	G/G	G/G
	Pontaleb	A/A	A/A	N	N	A/A	A/A	G/G	C/C	T/T	C/C	G/G	G/G	A/A	G/G	G/G
***Prunus-Amygdalus* hybrids and others**	AD 04-03	G/G	A/A	G/T	C/T	A/G	A/T	G/G	A/C	T/T	C/C	G/G	A/G	A/A	G/G	G/G
	AG 030104	G/G	A/C	G/G	C/T	A/A	A/A	A/G	A/C	T/T	C/T	G/G	A/G	G/G	A/A	G/G
	AG 030107	G/G	A/C	G/G	C/T	A/A	A/A	A/G	A/C	T/T	C/T	G/G	A/G	G/G	A/A	G/G
	AD 030112	G/G	A/C	G/G	N	A/A	A/A	A/G	A/C	T/T	C/T	G/G	A/G	A/G	A/G	G/G
	Citation CEAF	G/G	A/A	G/G	C/T	A/A	A/A	G/G	C/C	T/T	C/C	G/G	A/G	N	A/A	G/G
	Ishtara	G/G	A/A	G/G	T/T	A/A	A/A	N	A/C	T/T	C/C	C/C	A/A	G/G	A/A	G/G
	Nanking cherry	G/G	C/C	N	T/T	A/A	A/A	G/G	C/C	T/T	C/C	N	G/G	A/A	G/G	G/G
	PADAC 04-01	G/G	A/A	G/T	C/T	A/A	A/T	A/G	A/C	T/T	C/C	G/G	A/G	A/G	A/G	G/G
	PADAC 99-05	G/G	A/A	G/G	C/T	A/A	A/A	A/G	A/C	T/T	C/C	G/G	A/G	G/G	A/A	G/G
	R R	G/G	A/A	G/G	C/T	A/A	A/A	A/G	C/C	T/T	C/C	G/G	A/G	G/G	A/A	T/T

	Pseudomolecule<br>Position<br>Gene<br>Location	Pp02<br>5372807<br>Prupe.2G046400<br>Exon	Pp02<br>5597860<br>Prupe.2G048600<br>Intron	Pp02<br>7757386<br>Prupe.2G061100<br>Exon	Pp02<br>16188362<br>Prupe.2G105000<br>Exon	Pp02<br>17820932<br>Prupe.2G120800<br>Exon	Pp02<br>22441824<br>Prupe.2G181600<br>Exon	Pp02<br>22767709<br>Prupe.2G186700<br>Exon	Pp02<br>23077245<br>Prupe.2G191400<br>Exon	Pp02<br>23359464<br>Prupe.2G196200<br>Exon	Pp02<br>24535441<br>Prupe.2G214200<br>Intron	Pp02<br>24600784<br>Prupe.2G215000<br>Exon	Pp02<br>25504865<br>Prupe.2G230500<br>Intron	Pp02<br>25504866<br>Prupe.2G230500<br>Intron	Pp02<br>27301463<br>Prupe.2G266700<br>Exon	Pp02<br>28208092<br>Prupe.2G285200<br>Exon
	**Reference**	C/C	T/T	G/G	C/C	C/C	G/G	G/G	G/G	T/T	A/A	A/A	G/G	T/T	G/G	T/T
	**Variation**	G/G	C/C	T/T	A/A	A/A	T/T	A/A	A/A	G/G	G/G	T/T	T/T	C/C	T/T	A/A
Amygdalus	Adafuel	C/G	T/T	G/G	C/C	C/C	G/G	G/G	G/G	T/T	A/A	A/A	G/G	C/T	G/G	T/T
	Adarcias	C/G	T/T	G/G	C/C	C/C	G/G	G/G	G/G	T/T	A/A	A/A	G/G	T/T	G/G	T/T
	AG 000605	C/G	T/T	G/G	C/C	C/C	G/G	G/G	G/G	T/T	A/A	A/A	G/G	T/T	G/G	T/T
	AG 020409	C/C	T/T	G/G	C/C	C/C	G/G	G/G	G/G	T/T	A/A	A/A	G/G	T/T	G/G	T/T
	AG 060103	C/C	T/T	G/G	C/C	C/C	G/G	G/G	G/G	T/T	A/A	A/A	G/G	T/T	G/G	T/T
	AG 060104	C/C	T/T	G/G	C/C	C/C	G/G	G/G	G/G	T/T	A/A	A/A	G/G	T/T	G/G	T/T
	Cadaman	C/G	T/T	G/G	C/C	C/C	G/G	G/G	G/G	T/T	A/A	A/A	G/G	T/T	G/G	T/T
	Carmel	C/G	T/T	G/G	C/C	C/C	G/G	N	G/G	T/T	N	A/A	G/G	C/C	G/G	T/T
	Flordaguard	C/C	T/T	G/G	C/C	C/C	G/G	G/G	G/G	T/T	A/A	A/A	G/G	T/T	G/G	T/T
	Garnem	C/G	T/T	G/G	C/C	C/C	G/G	G/G	G/G	T/T	A/A	A/A	G/G	C/T	G/G	T/T
	GF 557	C/G	T/T	G/G	C/C	C/C	G/G	G/G	G/G	T/T	A/A	A/A	G/G	C/T	G/G	T/T
	GF 677	C/G	T/T	G/G	C/C	C/C	G/G	G/G	G/G	T/T	A/A	A/A	G/G	C/T	G/G	T/T
	Hansen 2168	C/G	T/T	G/G	C/C	C/C	G/G	G/G	G/G	T/T	A/A	A/A	G/G	C/T	G/G	T/T
	Hansen 536	C/G	T/T	G/G	C/C	C/C	G/G	G/G	G/G	T/T	A/A	A/A	G/G	C/T	G/G	T/T
	Ibdes 1	C/G	T/T	G/G	C/C	C/C	G/G	G/G	G/G	T/T	A/A	A/A	G/G	C/T	G/G	T/T
	Nemaguard m1	C/C	T/T	G/G	C/C	C/C	G/G	G/G	G/G	T/T	A/A	A/A	G/G	T/T	G/G	T/T
	Nemared	C/C	T/T	G/G	C/C	C/C	G/G	G/G	G/G	T/T	A/A	A/A	G/G	T/T	G/G	T/T
	PADAC 99-02	C/G	T/T	G/G	C/C	C/C	G/G	G/G	G/G	T/T	A/A	A/A	G/G	C/T	G/G	T/T
	Pomona	C/G	N	G/G	C/C	C/C	G/G	G/G	G/G	T/T	A/A	A/A	G/G	T/T	G/G	T/T
	R 40	C/G	T/T	G/G	C/C	C/C	G/G	G/G	G/G	T/T	A/A	A/A	G/G	C/T	G/G	T/T
	R 70	C/G	T/T	G/G	C/C	C/C	G/G	G/G	G/G	T/T	A/A	A/A	G/G	C/T	G/G	T/T
	R 90	C/G	T/T	G/G	C/C	C/C	G/G	G/G	G/G	T/T	A/A	A/A	G/G	C/T	G/G	T/T
	Tamarite de Litera	C/G	T/T	G/G	C/C	C/C	G/G	G/G	G/G	T/T	A/A	A/A	G/G	C/T	G/G	T/T
	Tauste 7	C/G	T/T	G/G	C/C	C/C	G/G	G/G	G/G	T/T	A/A	A/A	G/G	C/T	G/G	T/T
	Titan × Nemared	C/G	T/T	G/G	C/C	C/C	G/G	G/G	G/G	T/T	A/A	A/A	G/G	C/T	G/G	T/T
Prunus	Adara CSIC	C/G	C/C	G/T	A/C	A/C	G/G	A/A	G/G	T/T	A/G	T/T	T/T	T/T	G/T	T/T
	Ademir	C/C	C/C	G/G	C/C	A/C	G/G	A/A	G/G	T/T	A/G	T/T	T/T	T/T	G/T	A/T
	Angeleno	C/G	C/C	G/G	C/C	A/C	G/G	A/A	G/G	T/T	A/A	T/T	T/T	T/T	G/T	T/T
	Larry Ann	C/G	C/C	G/G	C/C	C/C	G/G	A/A	G/G	T/T	A/A	T/T	T/T	T/T	G/T	A/T
	Mariana 2624 CSIC	C/G	C/C	G/G	C/C	C/C	G/G	A/A	G/G	T/T	A/A	T/T	T/T	T/T	T/T	A/T
	Myrobalan 2201	C/G	C/C	G/G	C/C	A/C	G/G	A/A	A/G	T/T	A/G	T/T	T/T	T/T	T/T	T/T
	Myrobalan 2261	C/G	C/C	G/G	C/C	C/C	G/G	A/A	A/G	T/T	A/A	T/T	T/T	T/T	T/T	T/T
	Myrobalan 713AD	C/G	C/C	G/G	C/C	C/C	G/G	A/A	G/G	T/T	A/G	T/T	T/T	T/T	T/T	T/T
	Myrobalan B	C/G	C/C	G/G	C/C	A/C	G/G	A/A	A/G	T/T	A/G	T/T	T/T	T/T	T/T	T/T
	Myrobalan GF3-1	C/G	C/C	G/G	C/C	A/C	G/G	A/A	G/G	T/T	A/A	T/T	T/T	T/T	G/T	A/T
	Myrobalan m2	C/G	C/C	G/G	C/C	C/C	G/G	A/A	A/G	T/T	G/G	T/T	T/T	T/T	T/T	T/T
	Myrocal	C/G	C/C	G/G	C/C	A/C	G/G	A/A	G/G	T/T	A/A	T/T	T/T	T/T	T/T	T/T
	P1079	C/G	N	G/G	C/C	C/C	G/G	A/A	A/G	T/T	A/A	T/T	T/T	T/T	T/T	T/T
	P2175	C/G	C/C	G/T	C/C	A/C	G/G	A/A	A/G	T/T	A/A	T/T	T/T	T/T	G/T	A/T
	R 20	N	C/C	G/G	C/C	A/C	G/G	A/A	A/G	T/T	A/A	T/T	T/T	T/T	G/T	T/T
Cerasus	Bing	C/C	C/C	G/G	A/C	N	T/T	A/A	G/G	G/G	A/A	A/A	G/G	T/T	G/G	T/T
	Maxma 14	C/C	C/C	G/G	A/C	C/C	T/T	A/A	G/G	G/G	A/A	A/A	G/G	T/T	G/G	T/T
	Maxma 60	C/C	C/C	G/G	A/C	C/C	T/T	A/A	G/G	G/G	A/A	A/A	G/G	T/T	G/G	T/T
	Mazzard F12/1	C/C	C/C	G/G	A/C	N	T/T	A/A	G/G	G/G	A/A	A/A	G/G	T/T	G/G	T/T
	Pontaleb	C/G	C/C	G/G	C/C	C/C	T/T	A/A	G/G	G/G	A/A	A/A	G/G	T/T	G/G	T/T
***Prunus-Amygdalus* hybrids and others**	AD 04-03	C/G	T/T	G/T	C/C	C/C	G/G	G/G	G/G	T/T	A/G	A/T	G/T	T/T	G/T	T/T
	AG 030104	C/C	C/T	G/G	C/C	C/C	G/G	G/G	G/G	T/T	A/A	A/T	G/T	T/T	G/T	T/T
	AG 030107	C/C	C/T	G/G	C/C	C/C	G/G	G/G	A/G	T/T	A/A	A/T	G/T	T/T	G/T	T/T
	AD 030112	C/C	C/C	G/T	C/C	C/C	G/G	G/G	G/G	T/T	A/G	A/T	G/T	T/T	T/T	T/T
	Citation CEAF	N	T/T	G/G	C/C	C/C	G/G	A/G	G/G	T/T	A/A	T/T	G/T	T/T	G/T	T/T
	Ishtara	C/C	C/C	G/G	C/C	C/C	G/G	A/G	G/G	T/T	A/A	T/T	G/T	T/T	G/T	A/T
	Nanking cherry	C/C	C/C	G/G	C/C	C/C	G/G	N	G/G	T/T	A/A	A/A	T/T	T/T	G/G	A/A
	PADAC 04-01	C/G	T/T	G/T	C/C	C/C	G/G	G/G	G/G	T/T	A/G	A/T	G/T	T/T	G/T	T/T
	PADAC 99-05	C/G	T/T	G/T	A/C	C/C	G/G	A/G	G/G	T/T	A/A	A/T	G/T	T/T	T/T	T/T
	R R	C/G	C/T	G/G	C/C	C/C	G/G	G/G	G/G	T/T	A/A	T/T	G/T	T/T	G/T	T/T

	Pseudomolecule	Pp02	Pp02	Pp02	Pp02	Pp03	Pp03	Pp03	Pp03	Pp03	Pp03	Pp03	Pp03	Pp03	Pp03	Pp03
	**Position**	29118951	29778175	29786530	30052808	5152153	6026685	18483519	19659004	20034786	20242526	20710092	21042678	22341972	22395801	22712692
	**Gene**	Prupe.2G305600	Prupe.2G318400	Prupe.2G318700	Prupe.2G325000	Prupe.3G071800	Prupe.3G081000	Prupe.3G165800	Prupe.3G180700	Prupe.3G185800	Prupe.3G189300	Prupe.3G196300	Prupe.3G202400	Prupe.3G223400	Prupe.3G224500	Prupe.3G228000
	**Location**	Exon	Exon	Exon	Exon	Exon	Exon	Exon	Exon	Exon	Exon	Intron	Exon	Exon	Exon	Exon
	**Reference**	C/C	G/G	T/T	T/T	G/G	C/C	C/C	A/A	G/G	C/C	C/C	A/A	C/C	T/T	T/T
	**Variation**	T/T	A/A	G/G	G/G	T/T	A/A	G/G	T/T	T/T	A/A	A/A	T/T	T/T	A/A	C/C
Amygdalus	Adafuel	C/C	G/G	T/T	T/T	G/G	C/C	C/C	A/A	G/G	C/C	A/C	A/A	C/C	T/T	T/T
	Adarcias	C/C	G/G	T/T	T/T	G/G	C/C	C/C	A/A	G/G	C/C	C/C	N	T/T	T/T	T/T
	AG 000605	C/C	G/G	T/T	T/T	G/G	C/C	C/C	A/A	G/G	C/C	A/A	A/A	C/C	T/T	T/T
	AG 020409	C/C	G/G	T/T	T/T	G/G	C/C	C/C	A/A	G/G	C/C	A/C	A/A	C/T	T/T	T/T
	AG 060103	C/C	G/G	T/T	T/T	G/G	C/C	C/C	A/A	G/G	C/C	A/A	A/A	C/C	T/T	T/T
	AG 060104	C/C	G/G	T/T	T/T	G/G	C/C	C/C	A/A	G/G	C/C	A/A	A/A	C/C	T/T	T/T
	Cadaman	C/C	G/G	T/T	T/T	G/G	C/C	C/C	A/A	G/G	C/C	A/A	A/A	C/C	T/T	T/T
	Carmel	T/T	G/G	T/T	N	G/G	C/C	C/C	A/A	G/G	C/C	C/C	A/A	T/T	T/T	T/T
	Flordaguard	C/C	G/G	T/T	T/T	G/G	C/C	C/C	A/A	G/G	C/C	A/A	A/A	C/C	T/T	T/T
	Garnem	C/T	G/G	T/T	T/T	G/G	C/C	C/C	A/A	G/G	C/C	A/C	A/A	C/C	T/T	T/T
	GF 557	C/T	G/G	T/T	T/T	G/G	C/C	C/C	A/A	G/G	C/C	A/C	A/A	C/T	T/T	T/T
	GF 677	C/C	G/G	T/T	T/T	G/G	C/C	C/C	A/A	G/G	C/C	A/C	A/A	C/T	T/T	T/T
	Hansen 2168	C/T	G/G	T/T	T/T	G/G	C/C	C/C	A/A	G/G	C/C	A/C	A/A	C/T	T/T	T/T
	Hansen 536	C/T	G/G	T/T	T/T	G/G	C/C	C/C	A/A	G/G	C/C	A/C	A/A	C/T	T/T	T/T
	Ibdes 1	C/T	G/G	T/T	T/T	G/G	C/C	C/C	A/A	G/G	C/C	A/C	A/A	C/T	T/T	T/T
	Nemaguard m1	C/C	G/G	T/T	T/T	G/G	C/C	C/C	A/A	G/G	C/C	A/A	A/A	C/C	T/T	T/T
	Nemared	C/C	G/G	T/T	T/T	G/G	C/C	C/C	A/A	G/G	C/C	A/A	A/A	C/C	T/T	T/T
	PADAC 99-02	C/T	G/G	T/T	T/T	G/G	C/C	C/C	A/A	G/G	C/C	A/C	A/A	C/T	T/T	T/T
	Pomona	C/C	G/G	T/T	T/T	G/G	C/C	C/C	A/A	G/G	C/C	A/A	A/A	C/C	T/T	T/T
	R 40	C/T	G/G	T/T	T/T	G/G	C/C	C/C	A/A	G/G	C/C	A/C	A/A	C/C	T/T	T/T
	R 70	C/T	G/G	T/T	T/T	G/G	C/C	C/C	A/A	G/G	C/C	A/A	A/A	C/C	T/T	T/T
	R 90	C/T	G/G	T/T	T/T	G/G	C/C	C/C	A/A	G/G	C/C	A/C	A/A	C/C	T/T	T/T
	Tamarite de Litera	C/T	G/G	T/T	T/T	G/G	C/C	C/C	A/A	G/G	C/C	A/C	A/A	C/T	T/T	T/T
	Tauste 7	C/C	G/G	T/T	T/T	G/G	C/C	C/C	A/A	G/G	C/C	A/C	A/A	C/C	T/T	T/T
	Titan × Nemared	C/T	G/G	T/T	T/T	G/G	C/C	C/C	A/A	G/G	C/C	A/C	A/A	C/C	T/T	T/T
Prunus	Adara CSIC	C/C	A/A	T/T	T/T	T/T	C/C	C/G	A/T	G/G	C/C	C/C	T/T	C/C	T/T	C/T
	Ademir	C/C	A/A	T/T	T/T	T/T	A/C	C/G	A/T	G/G	C/C	C/C	T/T	C/C	T/T	C/C
	Angeleno	C/C	A/A	G/G	T/T	T/T	C/C	C/G	A/T	G/G	C/C	C/C	T/T	C/C	T/T	C/T
	Larry Ann	C/C	A/A	G/G	T/T	T/T	C/C	C/G	A/A	G/G	A/C	C/C	T/T	C/C	T/T	C/T
	Mariana 2624 CSIC	C/C	A/A	G/G	T/T	T/T	C/C	C/G	A/A	G/G	A/C	C/C	T/T	C/C	T/T	C/T
	Myrobalan 2201	C/C	A/A	T/T	T/T	T/T	C/C	C/C	A/T	G/G	C/C	C/C	T/T	C/C	T/T	C/C
	Myrobalan 2261	C/C	A/A	T/T	T/T	T/T	C/C	C/C	A/T	G/G	C/C	C/C	T/T	C/C	T/T	C/C
	Myrobalan 713AD	C/C	A/A	G/T	T/T	T/T	C/C	C/C	A/T	G/G	C/C	C/C	T/T	C/C	T/T	C/T
	Myrobalan B	C/C	A/A	G/G	T/T	T/T	C/C	C/G	A/T	G/G	C/C	C/C	T/T	C/C	T/T	C/C
	Myrobalan GF3-1	C/C	A/A	T/T	N	T/T	C/C	C/G	T/T	G/G	C/C	C/C	T/T	C/C	T/T	T/T
	Myrobalan m2	C/C	A/A	G/T	T/T	T/T	C/C	C/G	T/T	G/G	C/C	C/C	T/T	C/C	T/T	C/T
	Myrocal	C/C	A/A	T/T	T/T	T/T	C/C	C/C	T/T	G/G	C/C	C/C	T/T	C/C	T/T	C/T
	P1079	C/C	A/A	T/T	T/T	T/T	A/C	C/G	T/T	G/G	C/C	C/C	T/T	C/C	T/T	C/T
	P2175	C/C	A/A	T/T	T/T	T/T	C/C	C/G	A/T	G/G	N	C/C	T/T	C/C	T/T	C/C
	R 20	C/C	A/A	G/T	T/T	T/T	C/C	C/G	A/T	G/G	C/C	C/C	A/T	C/C	T/T	T/T
Cerasus	Bing	C/C	A/A	G/G	G/G	T/T	C/C	N	A/T	T/T	C/C	C/C	A/A	C/C	A/A	T/T
	Maxma 14	C/C	A/A	G/G	G/G	T/T	C/C	C/C	T/T	T/T	A/C	C/C	A/A	C/C	A/T	T/T
	Maxma 60	C/C	A/A	G/G	G/G	T/T	C/C	C/C	T/T	T/T	A/C	C/C	A/A	C/C	A/T	T/T
	Mazzard F12/1	C/C	A/A	G/G	G/G	T/T	C/C	N	T/T	T/T	C/C	C/C	A/A	C/C	A/A	T/T
	Pontaleb	C/C	A/A	G/G	G/G	T/T	C/C	C/C	T/T	T/T	A/C	N	N	C/C	T/T	T/T
***Prunus-Amygdalus* hybrids and others**	AD 04-03	C/C	A/G	T/T	T/T	G/T	N	C/C	A/T	G/G	C/C	C/C	A/T	C/C	T/T	C/T
	AG 030104	C/C	A/G	G/T	T/T	G/T	A/C	C/C	A/A	G/G	C/C	A/C	A/T	C/C	T/T	T/T
	AG 030107	C/C	A/G	G/T	T/T	G/T	C/C	C/C	A/A	G/G	C/C	A/C	A/T	C/C	T/T	T/T
	AD 030112	C/C	A/G	T/T	T/T	G/T	A/C	C/C	A/A	G/G	C/C	A/C	A/T	C/C	T/T	T/T
	Citation CEAF	C/C	A/G	G/T	T/T	G/T	A/C	C/C	A/A	G/G	C/C	A/C	A/T	C/C	T/T	T/T
	Ishtara	C/C	A/A	G/T	T/T	T/T	C/C	C/G	A/T	G/G	C/C	C/C	T/T	C/C	T/T	T/T
	Nanking cherry	C/C	A/A	G/G	N	T/T	N	C/C	N	G/G	C/C	C/C	N	C/C	T/T	T/T
	PADAC 04-01	C/C	A/G	T/T	T/T	G/T	C/C	C/G	A/A	G/G	C/C	C/C	A/T	C/C	T/T	T/T
	PADAC 99-05	C/C	A/G	T/T	T/T	G/T	C/C	C/C	A/T	G/G	C/C	C/C	T/T	C/C	T/T	N
	R R	C/C	A/G	T/T	T/T	G/T	A/C	C/C	A/T	G/G	C/C	A/C	A/T	C/C	T/T	C/T

	Pseudomolecule	Pp03	Pp03	Pp03	Pp03	Pp03	Pp03	Pp03	Pp04	Pp04	Pp04	Pp04	Pp04	Pp04	Pp04	Pp04
	**Position**	25578377	25784195	26188576	26460349	26845232	26952573	27263012	478027	3230538	4588318	4800787	12724300	13355976	16671331	18519647
	**Gene**	Prupe.3G279500	Prupe.3G284300	Prupe.3G292500	Prupe.3G299100	Prupe.3G308000	Prupe.3G310100	Prupe.3G315900	Prupe.4G009700	Prupe.4G066900	Prupe.4G091900	Prupe.4G095400	Prupe.4G204300	Prupe.4G213800	Prupe.4G249300	Prupe.4G258800
	**Location**	Exon	Exon	Exon	Exon	Exon	Intron	Exon	Exon	Intron	Exon	Exon	Intron	Exon	Intron	5'UTR
	**Reference**	A/A	A/A	T/T	C/C	C/C	A/A	C/C	T/T	C/C	C/C	G/G	A/A	C/C	T/T	G/G
	**Variation**	C/C	T/T	A/A	T/T	A/A	C/C	T/T	A/A	A/A	A/A	T/T	G/G	A/A	C/C	A/A
Amygdalus	Adafuel	A/A	A/A	T/T	C/C	C/C	A/A	C/T	T/T	C/C	C/C	G/T	A/G	C/C	T/T	G/G
	Adarcias	A/A	A/A	T/T	C/C	C/C	A/C	T/T	T/T	C/C	C/C	G/T	A/G	C/C	T/T	G/G
	AG 000605	A/A	A/A	T/T	C/C	C/C	A/A	C/C	T/T	C/C	C/C	G/G	A/G	C/C	T/T	G/G
	AG 020409	A/A	A/T	T/T	C/C	C/C	A/C	C/T	T/T	C/C	C/C	G/G	G/G	C/C	T/T	G/G
	AG 060103	A/A	A/A	T/T	C/C	C/C	A/A	C/C	T/T	C/C	C/C	G/G	G/G	C/C	T/T	G/G
	AG 060104	A/A	A/A	T/T	C/C	C/C	A/A	C/C	T/T	C/C	C/C	G/G	G/G	C/C	T/T	G/G
	Cadaman	A/A	A/A	T/T	C/C	C/C	A/A	C/C	T/T	C/C	C/C	G/G	A/A	C/C	T/T	G/G
	Carmel	A/A	T/T	T/T	C/C	C/C	C/C	T/T	T/T	C/C	C/C	T/T	A/A	C/C	T/T	G/G
	Flordaguard	A/A	A/A	T/T	C/C	C/C	A/A	C/C	T/T	C/C	C/C	G/G	G/G	C/C	T/T	G/G
	Garnem	A/A	A/A	T/T	C/C	C/C	A/A	C/T	T/T	C/C	C/C	G/T	A/G	C/C	T/T	G/G
	GF 557	A/A	A/T	T/T	C/C	C/C	A/C	C/T	T/T	C/C	C/C	G/G	A/G	C/C	T/T	G/G
	GF 677	A/A	A/A	T/T	C/C	C/C	A/A	C/T	T/T	C/C	C/C	G/T	A/A	C/C	T/T	G/G
	Hansen 2168	A/A	A/T	T/T	C/C	C/C	A/C	C/T	T/T	C/C	C/C	G/T	A/G	C/C	T/T	G/G
	Hansen 536	A/A	A/T	T/T	C/C	C/C	A/C	C/T	T/T	C/C	C/C	G/T	A/G	C/C	T/T	G/G
	Ibdes 1	A/A	A/A	T/T	C/C	C/C	A/C	C/T	T/T	C/C	C/C	G/T	A/G	C/C	T/T	G/G
	Nemaguard m1	A/A	A/A	T/T	C/C	C/C	A/A	C/C	T/T	C/C	C/C	G/G	G/G	C/C	T/T	G/G
	Nemared	A/A	A/A	T/T	C/C	C/C	A/A	C/C	T/T	C/C	C/C	G/G	G/G	C/C	T/T	G/G
	PADAC 99-02	A/A	A/T	T/T	C/C	C/C	A/C	C/T	T/T	C/C	C/C	G/G	A/G	C/C	T/T	G/G
	Pomona	A/A	A/A	T/T	C/C	C/C	A/A	C/C	T/T	C/C	C/C	G/G	G/G	C/C	T/T	G/G
	R 40	A/A	A/A	T/T	C/C	C/C	A/A	C/T	T/T	C/C	C/C	G/T	A/G	C/C	T/T	G/G
	R 70	A/A	A/A	T/T	C/C	C/C	A/A	C/C	T/T	C/C	C/C	G/T	A/A	C/C	T/T	G/G
	R 90	A/A	A/A	T/T	C/C	C/C	A/C	C/T	T/T	C/C	C/C	G/T	A/A	C/C	T/T	G/G
	Tamarite de Litera	A/A	A/A	T/T	C/C	C/C	A/C	C/T	T/T	C/C	C/C	G/T	A/A	C/C	T/T	G/G
	Tauste 7	A/A	A/A	T/T	C/C	C/C	A/C	N	T/T	C/C	C/C	G/G	A/G	C/C	T/T	G/G
	Titan × Nemared	A/A	A/T	T/T	C/C	C/C	A/C	C/T	T/T	C/C	C/C	G/T	A/G	C/C	T/T	G/G
Prunus	Adara CSIC	C/C	A/A	T/T	T/T	C/C	A/A	C/C	T/T	A/C	C/C	G/G	A/A	A/C	T/T	A/A
	Ademir	C/C	A/A	T/T	T/T	C/C	A/A	C/C	T/T	C/C	C/C	G/G	A/A	A/C	T/T	A/A
	Angeleno	C/C	A/A	T/T	T/T	C/C	A/A	C/C	T/T	A/C	C/C	G/G	A/A	A/A	T/T	A/A
	Larry Ann	C/C	A/A	T/T	T/T	C/C	A/A	C/C	T/T	C/C	C/C	G/G	A/A	A/C	T/T	A/A
	Mariana 2624 CSIC	C/C	A/A	T/T	T/T	C/C	A/A	C/C	T/T	C/C	C/C	G/G	A/A	A/C	T/T	A/A
	Myrobalan 2201	C/C	A/A	T/T	T/T	C/C	A/A	C/C	T/T	C/C	C/C	G/G	A/A	A/C	T/T	A/A
	Myrobalan 2261	C/C	A/A	T/T	T/T	C/C	A/A	C/C	T/T	C/C	C/C	G/G	A/A	A/A	T/T	A/A
	Myrobalan 713AD	C/C	A/A	T/T	T/T	C/C	A/A	C/C	T/T	C/C	C/C	G/G	A/A	A/A	T/T	A/A
	Myrobalan B	C/C	A/A	T/T	T/T	C/C	A/A	C/C	T/T	C/C	C/C	G/G	A/A	A/A	T/T	A/A
	Myrobalan GF3-1	C/C	A/A	T/T	T/T	C/C	A/A	C/C	T/T	C/C	C/C	G/G	A/A	A/A	T/T	A/A
	Myrobalan m2	C/C	A/A	T/T	T/T	C/C	A/A	C/C	T/T	C/C	C/C	G/G	A/A	A/A	T/T	A/A
	Myrocal	C/C	A/A	T/T	T/T	C/C	A/A	C/C	T/T	C/C	C/C	G/G	A/A	A/A	T/T	A/A
	P1079	C/C	A/A	T/T	T/T	C/C	A/A	C/C	T/T	C/C	C/C	G/G	A/A	A/A	T/T	A/A
	P2175	C/C	A/A	T/T	T/T	N	A/A	C/C	T/T	A/C	C/C	G/G	A/A	A/A	T/T	A/A
	R 20	C/C	A/A	T/T	T/T	A/C	A/A	C/C	T/T	C/C	C/C	G/G	A/A	C/C	T/T	A/A
Cerasus	Bing	C/C	A/A	A/A	N	N	N	C/C	A/A	C/C	A/A	G/G	A/A	C/C	C/C	G/G
	Maxma 14	C/C	A/A	A/A	C/C	A/A	A/A	C/C	A/T	C/C	A/A	G/G	A/A	C/C	C/C	G/G
	Maxma 60	C/C	A/A	A/A	C/C	A/A	A/A	C/C	A/A	C/C	A/A	G/G	A/A	C/C	C/C	G/G
	Mazzard F12/1	C/C	A/A	A/A	N	N	N	C/C	A/A	C/C	A/A	G/G	A/A	C/C	C/C	G/G
	Pontaleb	N	A/A	A/A	N	A/A	A/A	C/C	T/T	C/C	N	G/G	A/A	C/C	N	N
***Prunus-Amygdalus* hybrids and others**	AD 04-03	A/C	A/A	T/T	C/C	C/C	A/A	C/C	T/T	N	C/C	G/G	A/G	C/C	T/T	A/G
	AG 030104	A/C	A/A	T/T	C/C	C/C	A/A	C/C	T/T	C/C	C/C	G/G	A/G	C/C	T/T	A/G
	AG 030107	A/C	A/A	T/T	C/C	C/C	A/A	C/C	T/T	C/C	C/C	G/G	A/G	C/C	T/T	A/G
	AD 030112	A/C	A/A	T/T	C/C	C/C	A/A	C/C	T/T	C/C	C/C	G/G	A/G	A/C	T/T	A/G
	Citation CEAF	A/C	A/A	T/T	C/C	C/C	A/A	C/C	T/T	A/C	C/C	G/G	A/A	A/C	T/T	A/G
	Ishtara	C/C	A/A	T/T	T/T	C/C	A/A	C/C	T/T	A/C	C/C	G/G	A/A	A/C	T/T	A/G
	Nanking cherry	C/C	A/A	T/T	C/C	C/C	A/A	C/C	T/T	C/C	C/C	N	A/A	C/C	T/T	A/A
	PADAC 04-01	A/C	A/A	T/T	C/C	C/C	A/A	C/C	T/T	C/C	C/C	G/T	A/A	A/C	T/T	A/G
	PADAC 99-05	A/C	A/A	T/T	C/C	C/C	A/A	C/T	T/T	A/C	C/C	T/T	A/A	C/C	T/T	A/G
	R R	A/C	A/A	T/T	C/T	C/C	A/A	C/C	T/T	C/C	C/C	G/G	A/G	C/C	T/T	A/G

	Pseudomolecule	Pp04	Pp04	Pp05	Pp05	Pp05	Pp05	Pp05	Pp05	Pp05	Pp05	Pp05	Pp05	Pp05	Pp05	Pp05
	**Position**	21428422	24272831	2977555	2977592	2977655	9753025	11156615	11801339	12071378	12483740	13033824	13033825	13052939	13822461	14752314
	**Gene**	Prupe.4G269200	Prupe.4G283000	Prupe.5G026300	Prupe.5G026300	Prupe.5G026300	Prupe.5G085800	Prupe.5G105000	Prupe.5G115300	Prupe.5G121100	Prupe.5G128200	Prupe.5G138700	Prupe.5G138700	Prupe.5G139100	Prupe.5G153800	Prupe.5G173200
	**Location**	Exon	Exon	Intron	Intron	Intron	Exon	Exon	Exon	Exon	Exon	Exon	Exon	Intron	Exon	Exon
	**Reference**	G/G	A/A	C/C	C/C	G/G	C/C	T/T	C/C	T/T	C/C	T/T	A/A	T/T	C/C	G/G
	**Variation**	T/T	G/G	T/T	A/A	A/A	T/T	C/C	A/A	A/A	A/A	C/C	T/T	C/C	T/T	T/T
Amygdalus	Adafuel	G/G	A/A	C/C	C/C	G/G	C/C	T/T	C/C	A/T	C/C	T/T	A/A	T/T	C/C	G/G
	Adarcias	G/G	A/A	C/C	C/C	G/G	C/C	T/T	C/C	A/T	C/C	T/T	A/A	T/T	C/C	G/G
	AG 000605	G/G	A/A	C/C	C/C	G/G	C/C	T/T	C/C	A/A	C/C	T/T	A/A	T/T	C/C	G/G
	AG 020409	G/G	A/A	C/C	C/C	G/G	C/C	T/T	C/C	A/T	C/C	T/T	A/A	T/T	C/C	G/T
	AG 060103	G/G	A/A	C/C	C/C	G/G	C/C	T/T	C/C	A/A	C/C	T/T	A/A	T/T	C/C	G/G
	AG 060104	G/G	A/A	C/C	C/C	G/G	C/C	T/T	C/C	A/A	C/C	T/T	A/A	T/T	C/C	G/G
	Cadaman	G/G	A/A	C/C	C/C	G/G	C/C	T/T	C/C	A/T	C/C	T/T	A/A	T/T	C/C	G/G
	Carmel	G/G	A/A	C/C	C/C	N	C/C	T/T	C/C	T/T	C/C	T/T	A/A	T/T	C/C	G/T
	Flordaguard	G/G	A/A	C/C	C/C	G/G	C/C	T/T	C/C	A/A	C/C	T/T	A/A	T/T	C/C	G/G
	Garnem	G/G	A/A	C/C	C/C	G/G	C/C	T/T	C/C	A/T	C/C	T/T	A/A	T/T	C/C	G/T
	GF 557	G/G	A/A	C/C	C/C	G/G	C/C	T/T	C/C	A/T	C/C	T/T	A/A	T/T	C/C	G/G
	GF 677	G/G	A/A	C/C	C/C	G/G	C/C	T/T	N	A/T	C/C	T/T	A/A	T/T	C/C	G/G
	Hansen 2168	G/G	A/A	C/C	C/C	G/G	C/C	T/T	C/C	A/T	C/C	T/T	A/A	T/T	C/C	G/G
	Hansen 536	G/G	A/A	C/C	C/C	G/G	C/C	T/T	C/C	A/T	C/C	T/T	A/A	T/T	C/C	G/G
	Ibdes 1	G/G	A/A	C/C	C/C	G/G	C/C	T/T	C/C	A/T	C/C	T/T	A/A	T/T	C/C	G/G
	Nemaguard m1	G/G	A/A	C/C	C/C	G/G	C/C	T/T	C/C	A/A	C/C	T/T	A/A	T/T	C/C	G/G
	Nemared	G/G	A/A	C/C	C/C	G/G	C/C	T/T	C/C	A/A	C/C	T/T	A/A	T/T	C/C	G/G
	PADAC 99-02	G/G	A/A	C/C	C/C	G/G	C/C	T/T	C/C	A/T	C/C	T/T	A/A	T/T	C/C	G/G
	Pomona	G/G	A/A	C/C	C/C	G/G	C/C	T/T	C/C	A/A	C/C	T/T	A/A	T/T	C/C	G/G
	R 40	G/G	A/A	C/C	C/C	G/G	C/C	T/T	C/C	A/A	C/C	T/T	A/A	T/T	C/C	G/G
	R 70	G/G	A/A	C/C	C/C	G/G	C/C	T/T	C/C	A/T	C/C	T/T	A/A	T/T	C/C	G/G
	R 90	G/G	A/A	C/C	C/C	G/G	C/C	T/T	C/C	T/T	C/C	T/T	A/A	T/T	C/C	G/T
	Tamarite de Litera	G/G	A/A	C/C	C/C	G/G	C/C	T/T	C/C	A/A	C/C	T/T	A/A	T/T	C/C	G/T
	Tauste 7	G/G	A/A	C/C	C/C	G/G	C/C	T/T	C/C	A/T	C/C	T/T	A/A	T/T	C/C	G/G
	Titan × Nemared	G/G	A/A	C/C	C/C	G/G	C/C	T/T	C/C	A/T	C/C	T/T	A/A	T/T	C/C	G/G
Prunus	Adara CSIC	G/T	G/G	T/T	C/C	A/A	T/T	T/T	A/A	T/T	A/C	T/T	T/T	T/T	T/T	G/G
	Ademir	G/T	G/G	T/T	C/C	A/A	C/T	T/T	A/A	T/T	C/C	T/T	T/T	T/T	T/T	G/G
	Angeleno	G/G	G/G	C/T	C/C	A/A	C/C	T/T	A/A	T/T	C/C	T/T	T/T	T/T	T/T	G/G
	Larry Ann	G/G	G/G	C/T	C/C	A/A	C/T	T/T	A/A	T/T	A/C	T/T	T/T	T/T	C/T	G/G
	Mariana 2624 CSIC	G/G	G/G	C/T	C/C	A/A	C/T	T/T	A/A	T/T	A/C	T/T	T/T	T/T	C/T	G/G
	Myrobalan 2201	G/T	G/G	T/T	C/C	A/A	C/T	T/T	A/A	T/T	C/C	T/T	T/T	T/T	N	G/G
	Myrobalan 2261	G/T	G/G	T/T	C/C	A/A	C/T	T/T	A/A	T/T	A/C	N	N	T/T	T/T	G/G
	Myrobalan 713AD	G/T	G/G	T/T	C/C	A/A	C/C	T/T	A/A	T/T	A/C	T/T	T/T	T/T	T/T	G/G
	Myrobalan B	G/G	G/G	T/T	C/C	A/A	C/C	T/T	A/A	T/T	A/C	T/T	T/T	T/T	T/T	G/G
	Myrobalan GF3-1	G/T	G/G	T/T	C/C	A/A	C/C	T/T	A/A	T/T	C/C	T/T	T/T	T/T	T/T	G/G
	Myrobalan m2	G/G	G/G	T/T	C/C	A/A	C/T	T/T	A/A	T/T	A/C	T/T	T/T	T/T	T/T	G/G
	Myrocal	G/G	G/G	T/T	C/C	A/A	C/T	T/T	A/A	T/T	C/C	T/T	T/T	T/T	T/T	N
	P1079	G/G	G/G	T/T	C/C	A/A	T/T	T/T	A/A	T/T	A/C	T/T	T/T	T/T	T/T	G/G
	P2175	G/T	G/G	T/T	C/C	A/A	C/T	T/T	A/A	T/T	C/C	T/T	T/T	T/T	T/T	G/G
	R 20	G/T	G/G	C/T	C/C	A/A	C/T	T/T	A/A	T/T	C/C	T/T	T/T	T/T	C/T	G/G
Cerasus	Bing	G/G	A/A	C/C	A/A	G/G	C/C	C/C	C/C	T/T	A/C	C/C	A/A	C/C	C/C	G/G
	Maxma 14	G/G	G/G	C/C	A/C	G/G	C/C	C/C	C/C	T/T	C/C	C/C	A/A	C/C	C/C	G/G
	Maxma 60	G/G	A/A	C/C	A/C	G/G	C/C	C/C	C/C	T/T	C/C	C/C	A/A	C/C	C/T	G/G
	Mazzard F12/1	G/G	A/A	C/C	A/A	G/G	C/C	C/C	C/C	T/T	C/C	C/C	A/A	C/C	C/T	G/G
	Pontaleb	G/G	N	C/C	C/C	G/G	C/C	C/C	N	T/T	C/C	C/C	A/A	C/C	C/C	G/G
***Prunus-Amygdalus* hybrids and others**	AD 04-03	G/G	A/G	C/T	C/C	A/G	C/T	T/T	A/C	A/T	A/C	T/T	A/T	T/T	C/C	T/T
	AG 030104	G/T	A/G	C/C	C/C	A/G	C/T	T/T	A/C	A/T	C/C	T/T	A/T	T/T	C/C	G/G
	AG 030107	G/G	A/G	C/C	C/C	A/G	C/T	T/T	A/C	A/T	C/C	T/T	N	T/T	C/C	G/G
	AD 030112	G/G	A/G	C/C	C/C	A/G	C/C	T/T	A/C	A/T	A/C	T/T	A/A	T/T	C/T	G/G
	Citation CEAF	G/G	A/G	C/T	C/C	A/G	C/C	T/T	A/C	A/T	C/C	T/T	A/T	T/T	C/C	G/G
	Ishtara	G/G	A/G	C/T	C/C	A/G	C/C	T/T	A/C	A/T	A/C	T/T	A/A	T/T	C/T	G/G
	Nanking cherry	G/G	G/G	C/C	C/C	G/G	C/C	T/T	A/A	T/T	A/C	T/T	A/A	T/T	C/C	G/G
	PADAC 04-01	G/G	G/G	C/T	C/C	A/A	C/T	T/T	A/C	T/T	A/C	T/T	A/T	T/T	C/C	T/T
	PADAC 99-05	G/G	G/G	C/T	C/C	A/G	C/T	T/T	A/C	A/T	A/C	T/T	A/A	T/T	C/T	G/G
	R R	G/G	A/G	C/T	C/C	A/G	C/C	T/T	A/C	A/T	C/C	T/T	A/T	T/T	C/C	G/G

	**Pseudomolecule**	Pp05	Pp06	Pp06	Pp06	Pp06	Pp06	Pp06	Pp06	Pp06	Pp06	Pp06	Pp06	Pp06	Pp06	Pp07
	**Position**	15163706	4064429	5294814	6198291	6626204	6776765	11614585	24074941	24262177	25612767	25865964	25865968	25907095	28053849	355429
	**Gene**	Prupe.5G181100	Prupe.6G058100	Prupe.6G077600	Prupe.6G090600	Prupe.6G095500	Prupe.6G097200	Prupe.6G143200	Prupe.6G242400	Prupe.6G245400	Prupe.6G269200	Prupe.6G273700	Prupe.6G273700	Prupe.6G274200	Prupe.6G313800	Prupe.7G003000
	**Location**	Exon	Intron	Exon	Exon	Exon	5'UTR	Exon	Exon	Exon	Intron	Exon	Exon	Exon	Intron	Exon
	**Reference**	T/T	G/G	C/C	A/A	G/G	C/C	G/G	G/G	T/T	G/G	A/A	C/C	C/C	T/T	A/A
	**Variation**	C/C	A/A	A/A	T/T	A/A	T/T	T/T	T/T	A/A	T/T	T/T	A/A	T/T	C/C	T/T
Amygdalus	Adafuel	T/T	G/G	A/C	A/T	G/G	C/C	G/G	G/T	T/T	G/G	A/A	C/C	C/C	C/C	A/A
	Adarcias	T/T	G/G	C/C	A/T	G/G	C/C	G/G	G/T	T/T	G/G	A/A	C/C	C/C	C/C	A/A
	AG 000605	T/T	A/G	C/C	A/A	G/G	C/C	G/G	G/G	T/T	G/G	A/A	C/C	C/C	T/T	A/A
	AG 020409	T/T	G/G	C/C	A/A	G/G	C/C	G/G	G/T	T/T	G/G	A/A	C/C	C/C	C/T	A/A
	AG 060103	T/T	G/G	C/C	A/A	G/G	C/C	G/G	T/T	T/T	G/G	A/A	C/C	C/C	T/T	A/A
	AG 060104	T/T	G/G	C/C	A/A	G/G	C/C	G/G	T/T	T/T	G/G	A/A	C/C	C/C	T/T	A/A
	Cadaman	T/T	G/G	C/C	A/A	G/G	C/C	G/G	G/G	T/T	G/G	A/A	C/C	C/C	C/T	A/A
	Carmel	T/T	G/G	N	T/T	G/G	C/C	G/G	G/G	T/T	G/G	A/A	C/C	C/C	T/T	A/A
	Flordaguard	T/T	G/G	C/C	A/A	G/G	C/C	G/G	T/T	T/T	G/G	A/A	C/C	C/C	T/T	A/A
	Garnem	T/T	A/G	A/C	A/T	G/G	C/C	G/G	G/G	T/T	G/G	A/A	C/C	C/C	T/T	A/A
	GF 557	T/T	G/G	A/C	A/T	G/G	C/C	G/G	G/G	T/T	G/G	A/A	C/C	C/C	C/C	A/A
	GF 677	T/T	G/G	A/C	A/T	G/G	C/C	G/G	G/G	T/T	G/G	A/A	C/C	C/C	C/T	A/A
	Hansen 2168	T/T	G/G	N	A/T	G/G	C/C	G/G	G/T	T/T	G/G	A/A	C/C	C/C	C/C	A/A
	Hansen 536	T/T	G/G	A/C	A/T	G/G	C/C	G/G	G/T	T/T	G/G	A/A	C/C	C/C	C/C	A/A
	Ibdes 1	T/T	G/G	A/C	A/T	G/G	C/C	G/G	G/G	T/T	G/G	A/A	C/C	C/C	C/C	A/A
	Nemaguard m1	T/T	A/A	C/C	A/A	G/G	C/C	G/G	G/G	T/T	G/G	A/A	C/C	C/C	T/T	A/A
	Nemared	T/T	A/A	C/C	A/A	G/G	C/C	G/G	G/G	T/T	G/G	A/A	C/C	C/C	C/T	A/A
	PADAC 99-02	T/T	G/G	A/C	A/T	G/G	C/C	G/G	G/G	T/T	G/G	A/A	C/C	C/C	C/C	A/A
	Pomona	T/T	G/G	C/C	A/A	G/G	C/C	G/G	T/T	T/T	G/G	A/A	C/C	C/C	C/C	A/A
	R 40	T/T	G/G	A/A	T/T	G/G	C/C	G/G	G/G	T/T	G/G	A/A	C/C	C/C	C/C	A/A
	R 70	T/T	G/G	C/C	A/A	G/G	C/C	G/G	G/G	T/T	G/G	A/A	C/C	C/C	T/T	A/A
	R 90	T/T	G/G	C/C	A/A	G/G	C/C	G/G	G/G	T/T	G/G	A/A	C/C	C/C	C/C	A/A
	Tamarite de Litera	T/T	G/G	A/C	A/T	G/G	C/C	G/G	G/T	T/T	G/G	A/A	C/C	C/C	C/C	A/A
	Tauste 7	T/T	G/G	A/C	A/T	G/G	C/C	G/G	G/G	T/T	G/G	A/A	C/C	C/C	T/T	A/A
	Titan × Nemared	T/T	G/G	A/C	A/T	G/G	C/C	G/G	G/G	T/T	G/G	A/A	C/C	C/C	T/T	A/A
Prunus	Adara CSIC	C/C	G/G	C/C	A/A	A/A	C/C	T/T	G/G	A/A	T/T	T/T	A/A	C/C	T/T	A/T
	Ademir	C/C	G/G	C/C	A/A	A/A	C/C	T/T	G/G	A/A	T/T	T/T	A/A	C/C	T/T	T/T
	Angeleno	C/C	G/G	C/C	A/A	A/A	C/C	T/T	G/G	A/A	T/T	T/T	A/A	C/C	T/T	A/A
	Larry Ann	C/T	G/G	C/C	A/A	A/G	C/C	T/T	G/G	A/A	T/T	T/T	A/A	T/T	T/T	A/A
	Mariana 2624 CSIC	C/T	G/G	C/C	A/A	A/G	C/C	T/T	G/G	A/A	T/T	T/T	A/A	T/T	T/T	A/A
	Myrobalan 2201	C/C	G/G	C/C	A/A	A/A	C/C	T/T	G/G	A/A	T/T	T/T	A/A	T/T	T/T	A/A
	Myrobalan 2261	C/C	G/G	C/C	A/A	A/A	C/C	T/T	G/G	A/A	T/T	T/T	A/A	C/T	T/T	A/A
	Myrobalan 713AD	C/C	G/G	C/C	A/A	A/A	C/C	T/T	G/G	A/A	T/T	T/T	A/A	C/C	T/T	A/T
	Myrobalan B	C/C	G/G	C/C	A/A	A/A	C/C	T/T	G/G	A/A	T/T	T/T	A/A	T/T	T/T	A/A
	Myrobalan GF3-1	C/C	G/G	C/C	A/A	A/A	C/C	T/T	G/G	A/A	T/T	T/T	A/A	C/T	T/T	A/T
	Myrobalan m2	C/C	G/G	C/C	A/A	A/A	C/C	T/T	G/G	A/A	T/T	T/T	A/A	T/T	T/T	A/A
	Myrocal	C/C	G/G	C/C	A/A	A/A	C/C	T/T	G/G	A/A	T/T	T/T	A/A	T/T	T/T	A/T
	P1079	C/C	G/G	C/C	A/A	A/A	C/C	T/T	G/G	A/A	T/T	T/T	A/A	T/T	T/T	A/T
	P2175	C/C	G/G	C/C	A/A	A/A	C/C	T/T	G/G	A/A	T/T	T/T	A/A	C/T	T/T	A/T
	R 20	C/C	G/G	C/C	A/A	A/A	C/C	T/T	G/G	A/A	T/T	T/T	A/A	C/T	T/T	A/T
Cerasus	Bing	T/T	G/G	N	A/A	G/G	N	G/G	G/G	A/A	N	A/A	C/C	C/C	T/T	A/A
	Maxma 14	T/T	G/G	A/A	N	G/G	T/T	G/G	G/G	A/A	T/T	A/A	C/C	C/C	T/T	A/A
	Maxma 60	T/T	G/G	A/A	N	G/G	T/T	G/G	G/G	A/A	T/T	A/A	C/C	C/C	T/T	A/A
	Mazzard F12/1	T/T	G/G	A/A	A/A	N	N	G/G	G/G	A/A	N	N	N	C/C	T/T	A/A
	Pontaleb	T/T	N	A/A	N	G/G	T/T	G/G	G/G	A/A	T/T	N	N	N	T/T	A/A
***Prunus-Amygdalus* hybrids and others**	AD 04-03	C/T	G/G	A/C	A/T	A/A	C/C	G/T	G/G	A/T	G/T	A/T	A/C	C/C	T/T	A/A
	AG 030104	C/T	G/G	C/C	A/A	A/G	C/C	G/T	G/T	A/T	G/T	A/T	A/C	C/T	T/T	A/A
	AG 030107	C/T	G/G	C/C	A/A	A/G	C/C	G/T	G/T	A/T	G/T	A/T	A/C	C/T	T/T	A/A
	AD 030112	C/T	G/G	C/C	A/A	A/G	C/C	G/T	G/T	A/T	G/T	A/T	A/C	C/T	T/T	A/A
	Citation CEAF	C/T	G/G	C/C	A/A	A/G	C/C	G/T	G/G	A/T	G/T	A/T	A/C	C/C	T/T	A/A
	Ishtara	C/C	G/G	C/C	A/A	A/G	C/C	G/T	G/G	A/A	T/T	T/T	A/A	T/T	T/T	A/A
	Nanking cherry	T/T	N	C/C	A/A	G/G	C/C	G/G	G/G	A/A	T/T	N	N	N	T/T	A/A
	PADAC 04-01	C/T	G/G	A/C	A/T	A/A	C/C	G/T	G/G	A/T	G/T	A/T	A/C	C/C	T/T	A/T
	PADAC 99-05	C/T	G/G	A/C	A/T	A/G	C/C	G/T	G/G	A/T	G/T	A/T	A/C	C/C	T/T	A/A
	R R	C/T	G/G	C/C	A/A	A/G	C/C	G/T	G/G	A/T	G/T	A/T	A/C	C/C	T/T	A/A

	Pseudomolecule	Pp07	Pp07	Pp07	Pp07	Pp07	Pp07	Pp07	Pp07	Pp07	Pp08	Pp08	Pp08	Pp08	Pp08	Pp08
	**Position**	1892280	2597831	3862125	8234392	12024911	14919262	18684932	20336314	22201422	2964780	12198711	13462058	14751752	16714708	16744784
	**Gene**	Prupe.7G013300	Prupe.7G017900	Prupe.7G024500	Prupe.7G046300	Prupe.7G085200	Prupe.7G129200	Prupe.7G199400	Prupe.7G232800	Prupe.7G270900	Prupe.8G031300	Prupe.8G088700	Prupe.8G103900	Prupe.8G121500	Prupe.8G156100	Prupe.8G156700
	**Location**	Intron	Exon	3'UTR	Exon	Exon	Intron	Intron	Exon	Intron	Exon	Exon	Exon	Exon	Exon	Intron
	**Reference**	C/C	G/G	G/G	G/G	C/C	G/G	A/A	A/A	G/G	G/G	A/A	C/C	C/C	T/T	C/C
	**Variation**	T/T	T/T	A/A	T/T	G/G	T/T	G/G	G/G	C/C	C/C	T/T	G/G	A/A	A/A	A/A
Amygdalus	Adafuel	C/C	G/G	G/G	G/G	C/G	G/G	A/A	A/A	G/G	G/G	A/A	C/C	C/C	T/T	A/A
	Adarcias	C/C	G/G	G/G	G/T	C/G	G/G	A/A	A/A	G/G	G/G	A/A	C/C	C/C	T/T	A/A
	AG 000605	C/C	G/G	G/G	G/G	C/C	G/G	A/A	A/A	G/G	G/G	A/A	C/C	C/C	T/T	A/A
	AG 020409	C/C	G/G	G/G	G/T	C/C	G/G	A/A	A/A	G/G	G/G	A/A	C/C	C/C	T/T	A/A
	AG 060103	C/C	G/G	G/G	G/G	C/C	G/G	A/A	A/A	G/G	G/G	A/A	C/C	C/C	T/T	A/A
	AG 060104	C/C	G/G	G/G	G/G	C/C	G/G	A/A	A/A	G/G	G/G	A/A	C/C	C/C	T/T	A/A
	Cadaman	C/C	G/G	G/G	G/G	C/C	G/G	A/A	A/A	G/G	G/G	A/A	C/C	C/C	T/T	A/C
	Carmel	C/C	G/G	G/G	G/T	C/G	G/G	A/A	A/A	G/G	G/G	A/A	C/C	C/C	T/T	N
	Flordaguard	C/C	G/G	G/G	G/G	C/C	G/G	A/A	A/A	G/G	G/G	A/A	C/C	C/C	T/T	A/A
	Garnem	C/C	G/G	G/G	G/T	C/G	G/G	A/A	A/A	G/G	G/G	A/A	C/C	C/C	T/T	A/A
	GF 557	C/C	G/G	G/G	G/G	C/G	G/G	A/A	A/A	G/G	G/G	A/A	C/C	C/C	T/T	A/A
	GF 677	C/C	G/G	G/G	G/G	C/C	G/G	A/A	A/A	G/G	G/G	A/A	C/C	C/C	T/T	A/A
	Hansen 2168	C/C	G/G	G/G	G/T	C/C	G/G	A/A	A/A	G/G	G/G	A/A	C/C	C/C	T/T	C/C
	Hansen 536	C/C	G/G	G/G	N	C/C	G/G	A/A	A/A	G/G	G/G	A/A	C/C	C/C	T/T	C/C
	Ibdes 1	C/C	G/G	G/G	G/G	C/G	G/G	A/A	A/A	G/G	G/G	A/A	C/C	C/C	T/T	A/A
	Nemaguard m1	C/C	G/G	G/G	G/G	C/C	G/G	A/A	A/A	G/G	G/G	A/A	C/C	C/C	T/T	A/A
	Nemared	C/C	G/G	G/G	G/G	C/C	G/G	A/A	A/A	G/G	G/G	A/A	C/C	C/C	T/T	A/A
	PADAC 99-02	C/C	G/G	G/G	G/G	C/G	G/G	A/A	A/A	G/G	G/G	A/A	C/C	C/C	T/T	A/A
	Pomona	C/C	G/G	G/G	G/G	C/C	G/G	A/A	A/A	G/G	G/G	A/A	C/C	C/C	T/T	A/A
	R 40	C/C	G/G	G/G	G/G	C/G	G/G	A/A	A/A	G/G	G/G	A/A	C/C	C/C	T/T	A/A
	R 70	C/C	G/G	G/G	G/G	C/C	G/G	A/A	A/A	G/G	G/G	A/A	C/C	C/C	T/T	A/C
	R 90	C/C	G/G	G/G	G/G	C/C	G/G	A/A	A/A	G/G	G/G	A/A	C/C	C/C	T/T	A/C
	Tamarite de Litera	C/C	G/G	G/G	G/G	C/C	G/G	A/A	A/A	G/G	G/G	A/A	C/C	C/C	T/T	A/A
	Tauste 7	C/C	G/G	G/G	G/G	C/G	G/G	A/A	A/A	G/G	G/G	A/A	C/C	C/C	T/T	A/A
	Titan × Nemared	C/C	G/G	G/G	G/T	C/G	G/G	A/A	A/A	G/G	G/G	A/A	C/C	C/C	T/T	A/A
Prunus	Adara CSIC	C/T	G/G	G/G	G/G	C/C	T/T	G/G	A/A	G/G	C/G	T/T	C/C	C/C	A/A	C/C
	Ademir	C/T	G/G	G/G	G/G	C/C	T/T	G/G	A/A	G/G	C/G	T/T	C/C	C/C	A/A	C/C
	Angeleno	C/C	G/G	G/G	G/G	C/C	G/T	G/G	A/A	N	G/G	T/T	C/C	C/C	A/A	C/C
	Larry Ann	C/T	G/G	G/G	G/G	C/C	G/T	G/G	A/A	G/G	G/G	A/T	C/C	C/C	A/A	C/C
	Mariana 2624 CSIC	C/T	G/G	G/G	G/G	C/C	G/T	G/G	A/A	G/G	G/G	A/T	C/C	C/C	A/A	C/C
	Myrobalan 2201	C/T	G/G	G/G	G/G	C/C	T/T	G/G	A/A	G/G	G/G	T/T	C/C	C/C	A/A	C/C
	Myrobalan 2261	C/T	G/G	G/G	G/G	C/C	T/T	G/G	A/A	G/G	G/G	T/T	C/C	C/C	A/A	C/C
	Myrobalan 713AD	C/T	G/G	G/G	G/G	C/C	T/T	G/G	A/A	G/G	C/G	T/T	C/C	C/C	A/A	C/C
	Myrobalan B	C/T	G/G	G/G	G/G	C/C	T/T	G/G	A/A	G/G	G/G	T/T	C/C	C/C	A/A	C/C
	Myrobalan GF3-1	C/T	G/G	G/G	G/G	C/C	G/T	G/G	A/A	G/G	G/G	T/T	C/C	C/C	A/A	C/C
	Myrobalan m2	C/T	G/G	G/G	G/G	C/C	T/T	G/G	A/A	G/G	G/G	T/T	N	C/C	A/A	C/C
	Myrocal	C/T	G/G	G/G	G/G	C/C	T/T	G/G	A/A	G/G	G/G	T/T	C/C	C/C	A/A	C/C
	P1079	C/T	G/G	G/G	G/G	C/C	T/T	G/G	A/A	G/G	G/G	T/T	C/C	C/C	A/A	C/C
	P2175	C/T	G/G	G/G	G/G	C/C	T/T	G/G	A/A	G/G	G/G	T/T	C/C	C/C	A/A	C/C
	R 20	C/T	G/G	G/G	G/G	C/C	T/T	G/G	A/A	G/G	C/G	A/T	C/C	C/C	A/A	C/C
Cerasus	Bing	C/C	T/T	A/G	G/G	C/C	N	A/A	G/G	C/C	G/G	A/A	G/G	A/A	A/A	N
	Maxma 14	C/C	G/T	A/A	G/G	C/C	G/G	A/A	A/G	C/C	G/G	A/A	C/G	A/A	A/A	C/C
	Maxma 60	C/C	G/T	A/A	G/G	C/C	G/G	A/A	A/G	C/C	G/G	A/A	C/G	A/A	A/A	C/C
	Mazzard F12/1	C/C	T/T	A/G	G/G	C/C	N	A/A	G/G	C/C	G/G	A/A	G/G	A/A	A/A	C/C
	Pontaleb	C/C	G/G	N	G/G	C/C	G/G	N	A/A	C/C	G/G	N	C/C	N	A/A	C/C
***Prunus-Amygdalus* hybrids and others**	AD 04-03	C/T	G/G	G/G	G/T	C/G	G/T	A/G	A/A	G/G	C/G	T/T	C/C	C/C	T/T	C/C
	AG 030104	C/C	G/G	G/G	G/G	C/C	G/G	A/G	A/A	G/G	G/G	A/T	C/C	C/C	T/T	A/C
	AG 030107	C/C	G/G	G/G	G/G	C/C	G/T	A/G	A/A	G/G	G/G	A/T	C/C	C/C	T/T	A/C
	AD 030112	C/C	G/G	G/G	G/G	C/C	G/G	A/G	A/A	G/G	G/G	A/T	C/C	C/C	T/T	A/C
	Citation CEAF	C/T	G/G	G/G	G/G	C/C	G/G	A/G	A/A	G/G	G/G	A/T	C/C	C/C	T/T	A/C
	Ishtara	C/T	G/G	G/G	G/G	C/C	G/T	A/G	A/A	G/G	C/G	T/T	C/C	C/C	A/A	C/C
	Nanking cherry	C/C	G/G	G/G	G/G	C/C	N	A/A	A/A	G/G	G/G	A/A	C/C	C/C	A/A	C/C
	PADAC 04-01	C/T	G/G	G/G	G/G	C/C	G/T	A/G	A/A	G/G	G/G	A/T	C/C	C/C	N	A/C
	PADAC 99-05	C/T	G/G	G/G	G/G	C/C	G/T	A/G	A/A	G/G	C/G	A/T	C/C	C/C	N	A/C
	R R	C/T	G/G	G/G	G/G	C/C	G/T	A/G	A/A	G/G	C/G	A/T	C/C	C/C	T/T	A/C

	Pseudomolecule	Pp08	Pp08	Pp08	Pp08	Pp08	Pp08	Pp08	Pp08
	**Position**	17720052	18250622	18484118	19316635	19568186	19568187	19922349	20765179
	**Gene**	Prupe.8G174100	Prupe.8G183500	Prupe.8G189000	Prupe.8G206200	Prupe.8G211800	Prupe.8G211800	Prupe.8G218300	Prupe.8G234400
	**Location**	Intron	3'UTR	Exon	Exon	Exon	Exon	Exon	Exon
	**Reference**	G/G	T/T	G/G	A/A	T/T	C/C	C/C	A/A
	**Variation**	A/A	G/G	A/A	G/G	A/A	A/A	A/A	T/T
Amygdalus	Adafuel	G/G	T/T	G/G	A/A	T/T	C/C	C/C	A/A
	Adarcias	G/G	T/T	G/G	A/A	T/T	C/C	C/C	A/A
	AG 000605	G/G	T/T	G/G	A/A	T/T	C/C	C/C	A/A
	AG 020409	G/G	T/T	G/G	A/A	T/T	C/C	C/C	A/A
	AG 060103	G/G	T/T	G/G	A/A	T/T	C/C	C/C	A/A
	AG 060104	G/G	T/T	G/G	A/A	T/T	C/C	C/C	A/A
	Cadaman	G/G	T/T	G/G	A/A	T/T	C/C	C/C	A/A
	Carmel	G/G	T/T	G/G	A/A	T/T	C/C	C/C	A/A
	Flordaguard	G/G	T/T	G/G	A/A	T/T	C/C	C/C	A/A
	Garnem	G/G	T/T	G/G	A/A	T/T	C/C	C/C	A/A
	GF 557	G/G	T/T	G/G	A/A	T/T	C/C	C/C	A/A
	GF 677	G/G	T/T	G/G	A/A	T/T	C/C	C/C	A/A
	Hansen 2168	N	T/T	G/G	A/A	T/T	C/C	C/C	A/A
	Hansen 536	N	T/T	G/G	A/A	T/T	C/C	C/C	A/A
	Ibdes 1	G/G	T/T	G/G	A/A	T/T	C/C	C/C	A/A
	Nemaguard m1	G/G	T/T	G/G	A/A	T/T	C/C	C/C	A/A
	Nemared	G/G	T/T	N	A/A	T/T	C/C	C/C	A/A
	PADAC 99-02	G/G	T/T	G/G	A/A	T/T	C/C	C/C	A/A
	Pomona	G/G	T/T	G/G	A/A	T/T	C/C	C/C	A/A
	R 40	G/G	T/T	G/G	A/A	T/T	C/C	C/C	A/A
	R 70	G/G	T/T	G/G	A/A	T/T	C/C	C/C	A/A
	R 90	G/G	T/T	G/G	A/A	T/T	C/C	C/C	A/A
	Tamarite de Litera	G/G	T/T	G/G	A/A	T/T	C/C	C/C	A/A
	Tauste 7	G/G	T/T	G/G	A/A	T/T	C/C	C/C	A/A
	Titan × Nemared	G/G	T/T	G/G	A/A	T/T	C/C	C/C	A/A
Prunus	Adara CSIC	A/G	T/T	G/G	A/A	A/A	A/A	C/C	T/T
	Ademir	A/G	T/T	G/G	A/A	A/A	A/A	C/C	T/T
	Angeleno	A/A	T/T	G/G	A/A	A/A	A/A	C/C	T/T
	Larry Ann	G/G	T/T	G/G	A/A	A/A	A/A	C/C	T/T
	Mariana 2624 CSIC	A/A	N	N	A/A	A/A	A/A	C/C	T/T
	Myrobalan 2201	G/G	T/T	G/G	A/A	A/A	A/A	C/C	T/T
	Myrobalan 2261	G/G	T/T	G/G	A/A	A/A	A/A	C/C	T/T
	Myrobalan 713AD	A/G	T/T	G/G	A/A	A/A	A/A	C/C	T/T
	Myrobalan B	G/G	T/T	G/G	A/A	A/A	A/A	C/C	T/T
	Myrobalan GF3-1	A/G	T/T	G/G	A/A	A/A	A/A	C/C	T/T
	Myrobalan m2	G/G	T/T	G/G	A/A	A/A	A/A	C/C	T/T
	Myrocal	A/G	T/T	G/G	A/A	A/A	A/A	C/C	T/T
	P1079	G/G	N	G/G	A/A	A/A	A/A	C/C	T/T
	P2175	G/G	T/T	G/G	A/A	A/A	A/A	C/C	T/T
	R 20	G/G	T/T	G/G	A/A	A/A	A/A	C/C	T/T
Cerasus	Bing	G/G	G/G	A/A	G/G	A/A	A/A	N	T/T
	Maxma 14	G/G	G/T	A/A	G/G	A/A	A/A	A/A	T/T
	Maxma 60	G/G	G/T	A/A	G/G	A/A	A/A	A/A	T/T
	Mazzard F12/1	G/G	G/G	A/A	G/G	A/A	A/A	N	T/T
	Pontaleb	A/G	T/T	N	N	N	N	A/A	T/T
***Prunus-Amygdalus* hybrids and others**	AD 04-03	G/G	T/T	G/G	A/A	A/T	A/C	C/C	A/T
	AG 030104	G/G	T/T	G/G	A/A	A/T	A/C	C/C	A/T
	AG 030107	G/G	T/T	G/G	A/A	A/T	A/C	C/C	A/T
	AD 030112	G/G	T/T	G/G	A/A	A/T	A/C	C/C	A/T
	Citation CEAF	G/G	T/T	G/G	A/A	A/T	A/C	C/C	A/T
	Ishtara	A/A	T/T	G/G	A/A	A/A	A/A	C/C	T/T
	Nanking cherry	G/G	T/T	G/G	A/A	N	N	C/C	T/T
	PADAC 04-01	A/G	T/T	G/G	A/A	A/T	A/C	C/C	A/T
	PADAC 99-05	G/G	N	G/G	A/A	A/T	A/C	C/C	A/T
	R R	G/G	T/T	G/G	A/A	A/T	A/C	C/C	A/T

N – Missing data
